# Comparison of Repeated Doses of Ivermectin Versus Ivermectin Plus Albendazole for the Treatment of Onchocerciasis: A Randomized, Open-label, Clinical Trial

**DOI:** 10.1093/cid/ciz889

**Published:** 2019-09-19

**Authors:** Linda Batsa Debrah, Ute Klarmann-Schulz, Jubin Osei-Mensah, Bettina Dubben, Kerstin Fischer, Yusif Mubarik, Nana Kwame Ayisi-Boateng, Arcangelo Ricchiuto, Rolf Fimmers, Peter Konadu, Jennifer Nadal, Barbara Gruetzmacher, Gary Weil, James W Kazura, Christopher L King, Alexander Y Debrah, Achim Hoerauf

**Affiliations:** 1 Kumasi Center for Collaborative Research, Kumasi, Ghana; 2 Department of Clinical Microbiology, School of Medicine and Dentistry, Kwame Nkrumah University of Science and Technology, Kumasi, Ghana; 3 Institute of Medical Microbiology, Immunology and Parasitology, University Hospital Bonn, Germany; 4 German Center for Infection Research, Bonn-Cologne site, Germany; 5 Institute for Medical Biometry, Informatics and Epidemiology, University Hospital Bonn, Germany; 6 Infectious Diseases Division, Department of Medicine, Washington University School of Medicine, St. Louis, Missouri; 7 University Hospital, , Kumasi, Ghana; 8 School of Medicine and Dentistry, Kwame Nkrumah University of Science and Technology, Kumasi, Ghana; 9 Center for Global Health and Diseases, Case Western Reserve University School of Medicine, Cleveland, Ohio; 10 Veterans Affairs Medical Center, Cleveland, Ohio; 11 Faculty for Allied Health Sciences, Kwame Nkrumah University of Science and Technology, Kumasi, Ghana

**Keywords:** onchocerciasis, therapy, ivermectin, albendazole, semiannual treatment

## Abstract

**Background:**

Improved treatment for onchocerciasis is needed to accelerate onchocerciasis elimination in Africa. Aiming to better exploit registered drugs, this study was undertaken to determine whether annual or semiannual treatment with ivermectin (IVM; 200 µg/kg) plus albendazole (ALB; 800 mg single dose) is superior to IVM alone.

**Methods:**

This trial was performed in Ghana and included 272 participants with microfilariae (MF), who were randomly assigned to 4 treatment arms: (1) IVM annually at 0, 12, and 24 months; (2) IVM semiannually at 0, 6, 12, 18, and 24 months; (3) IVM+ALB annually; or (4) IVM+ALB semiannually. Microfiladermia was determined pretreatment and at 6, 18, and 36 months. The primary outcome was the proportion of fertile and viable female worms in onchocercomata excised at 36 months.

**Results:**

Posttreatment nodule histology showed that 15/135 (11.1%), 22/155 (14.2%), 35/154 (22.7%), and 20/125 (16.0%) living female worms had normal embryogenesis in the IVM annual, IVM semiannual, IVM+ALB annual, and IVM+ALB semiannual groups, respectively (*P* = .1229). Proportions of dead worms also did not differ between the 4 groups (*P* = .9198). Proportions of patients without MF at 36 months (1 year after the last treatment) were 35/56 (63%) after annual IVM, 42/59 (71%) after semiannual IVM, 39/64 (61%) after annual IVM+ALB, and 43/53 (81%) after semiannual IVM+ALB.

**Conclusions:**

The combination treatment of IVM plus ALB was no better than IVM alone for sterilizing, killing adult worms, or achieving sustained MF clearance. However, semiannual treatment was superior to annual treatment for achieving sustained clearance of *Onchocerca volvulus* MF from the skin (*P* = .024).

**Clinical Trials Registration:**

ISRCTN50035143

## Teaser

This randomized controlled trial shows that albendazole plus ivermectin is no better for the treatment of onchocerciasis than ivermectin alone. Semiannual treatment was superior to annual treatment for suppressing microfilariae in the skin.

Onchocerciasis is a vector-borne nematode disease spread by black flies (*Simulium* species). While the adult worms that develop from transmitted third-stage larvae (L3) reside in subcutaneous nodules (onchocercomata) and do little harm, the microfilariae (MF) that are being released cause dermatitis when they reside in the skin and ocular lesions when they migrate into the eye [1].

Several developments have greatly improved the onchocerciasis situation since the 1970s. Vector control by the Onchocerciasis Control Programme in West Africa and mass drug administration (MDA) of Ivermectin (IVM; Mectizan) by the African Program for Onchocherciasis Control (ended 2015) have considerably reduced parasite infection intensities and onchocerciasis disease rates in many endemic countries [2]. With a smaller budget to cover 5 neglected tropical diseases (NTDs; lymphatic filariasis [LF], onchocerciasis, soil-transmitted helminths [STH], trachoma, schistosomiasis) that are amenable to preventive chemotherapy, the new Expanded Special Project for Elimination of NTDs [3] will have to rely on donated drugs, financial donations to cover operational costs, and endemic countries’ drug distribution programs for many more years.

While IVM has good activity against MF, in the doses commonly used for onchocerciasis control programs it does not kill *Onchocerca volvulus* adult worms, which have a reproductive life span of 12–14 years [4, 5]. Adult worms resume production of MF, which can lead to transmission of new onchocerciasis, within a few months after IVM treatment.

It is therefore widely accepted that new, IVM-complementing regimens are needed that are either macrofilaricidal or long-term sterilizing in order to speed up the elimination process if the milestones set by the London Declaration on NTDs for 2020 or the World Health Organization’s 2030 Sustainable Development Goals are to be met [6]. Repeated rounds of MDA with IVM alone may not be sufficient to eliminate the transmission of onchocerciasis in many African countries before 2050 [7].

Development of novel drugs is well under way, and several candidates are in early stages of clinical development (https://www.dndi.org/). However, it will be many years before any of these drugs can be widely used. This is also true to a lesser extent for moxidectin, a recently registered new drug that is superior to IVM for suppressing microfiladermia [8–10]. The Death to Onchocerciasis and Lymphatic Filariasis Project (https://dolf.wustl.edu/) was funded in 2010 to optimize therapy with existing drugs for elimination of LF and onchocerciasis, and not just disease control, in alignment with the new World Health Organization roadmap to 2030. Currently, ALB plus IVM is widely used in Africa for the elimination of LF, as well as in many areas co-endemic with onchocerciasis, and shows an excellent safety record. It is unknown whether ALB plus IVM has an added impact on onchocerciasis adult worm viability and sterility, compared to IVM alone. If so, this combination could be used in areas where onchocerciasis occurs without LF. ALB has been demonstrated to have embryotoxic effects in adult, female, *O. volvulus* worms [11, 12]. Therefore, the purpose of this study was to compare the effects of IVM+ALB with those of IVM alone for killing and/or sterilizing adult, female, *O. volvulus* worms and for clearing/suppressing skin MF.

## METHODS

### Study Population and Ethics Statement

The trial was registered at International Standard Randomised Controlled Trial Number (https://doi.org/10.1186/ISRCTN50035143) and approved by the Committee on Human Research, Publications and Ethics of the Kwame Nkrumah University of Science and Technology, Kumasi, Ghana; the Case Western Reserve University Institutional Review Board, Cleveland, Ohio; the Ethics Committee of the University Bonn, Germany; and the Ghana Food and Drugs Authority.

The randomized, controlled trial (RCT) was carried out in the Adansi South and Amansie Central Districts in the Ashanti Region. In these districts, according to official files, annual MDA with IVM had been carried out since 2009 (coverage 82–84%). Twice yearly, MDA had not been undertaken until the end of the trial. Volunteers with MF aged 18–60 years with at least 1 palpable onchocercoma but otherwise healthy were eligible. Exclusion criteria were: last IVM treatment <7 months ago, pregnancy, breastfeeding, having a serious medical illness, weight <40 kg, aspartate aminotransferase, alanine transaminase, gamma-glutamyltransferase >1.5 ULN, or significant glycosuria or proteinuria. Written or thumb printed informed consent was obtained from all individuals prior to enrollment.

### Study Design, Randomization, and Interventions

This trial was a randomized, open-label clinical trial with participants from 36 onchocerciasis-endemic communities with IVM-MDA since 2009.

Eligible volunteers were randomized to either:

Arm 1: IVM annual, which was an annual single dose of IVM at 200 µg/kg, given at 0, 12, and 24 months.
Arm 2: IVM semiannual, which was a semiannual single dose of IVM at 200 µg/kg, given at 0, 6, 12, 18, and 24 months.
Arm 3: IVM+ALB annual, which was an annual single dose of IVM at 200 µg/kg plus ALB at 800 mg, given at 0, 12. and 24 months.
Arm 4: IVM+ALB semiannual, which was a semiannual single dose of IVM at 200 µg/kg plus ALB at 800 mg, given at 0, 6, 12, 18, and 24 months.

Instead of the 150 µg/kg regularly used for onchocerciasis, a dose of IVM at 200 µg/kg was chosen, as it is commonly used for treating LF. The ALB dose of 800 mg is twice the normal dose usually administered for LF or STH.

Individuals in the annual arms received vitamin pills at 6 and 18 months. To ensure an equal number of IVM-naive patients in every treatment group, participants with a history of prior IVM intake were separately randomized.

The primary outcome of this trial was the percentage of fertile, female, adult worms in accessible nodules at 36 months following initiation of therapy. An alternating logistic regression analysis (GENMOD, SAS), following the closed testing procedure, was chosen for the analysis of the primary outcome, because it allows for correcting of the possible dependency of the observation on different worms in 1 patient. See Supplementary Figure 1 for details on power calculation/sample size estimation.

### Safety Monitoring of Study Participants

After the administration of study medication, an active assessment of adverse events was done for the first 3 days and passive assessments continued for another 4 days for all study participants

### Parasitological Assessment

**Figure 1.**
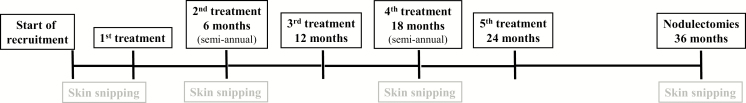
Study timeline, starting with the recruitment and ending with the surgical removal of the onchocercomata (nodulectomies) 36 months after the first treatment. In addition to the treatment time points, the figure also shows the time points when small skin biopsies (skin snipping) were taken to assess the microfilaria load in the skin.

We took 2 snips of 1–3 mg from skin over the superior iliac crests, using a corneoscleral punch (Holth), to determine skin MF loads at baseline and at 6, 18, and 36 months (Figure 1). Each snip was immersed in 100 µl of a 0.9% sodium chloride (NaCl) solution in a microtiter plate well. Snips were incubated overnight at room temperature to allow MF to emerge. The solution was then transferred onto a slide for microscopy. The snips were weighed using an analytical balance and the MF load was calculated as MF per mg of skin [13, 14].

### Histological Assessment

Nodulectomies were performed at 35.4 ± 0.9 months (range 34–36 months) after the first treatment and at 10.9 ± 0.4 (range 10–12) months after the last treatment. The nodules were fixed in 80% ethanol or a 4% phosphate-buffered formaldehyde solution. Samples were embedded in paraffin and sections of the nodules were stained with hematoxylin and eosin, cathepsin D-like lysosomal aspartic protease of *O. volvulus* for worm vitality, and Gomori’s method for iron [13]. At least 6 nodule sections were histologically assessed [15–17] by 2 assessors (B. D. and K. F.), who were blinded regarding treatment assignments. To ensure reliability, both assessors performed the analyses independently. Results from the independent assessments were entered in REDCap by double data entry (Research Electronic Data Capture) [18]. The 2 assessors met in person to review and resolve discrepancies in nodule readings.

### Data Collection at the Trial Sites

Paper-based case report forms were used at the trial sites. Subsequently, the data were entered by double data entry into REDCap, hosted at the University Hospital Bonn [18].

### Statistics

Analyses were done using SAS version 9.4 (SAS Institute Inc., Cary, NC) and SPSS (IBM SPSS Statistics 24, Armonk, NY). We used 2 data sets (per protocol, PP; intention to treat, ITT) to analyze the data. The ITT set includes all participants randomized to 1 of the 4 treatment arms who took the drugs at least once. This analysis set was also used to analyze the safety data. The PP set (subset of the ITT set) includes all patients who completed the treatment without any violations of the protocol and were present for nodulectomies at 36 month. The ITT set was used for the primary analysis of all parameters. The PP set was used to confirm the ITT results.

Baseline data were analyzed using an analysis of variance for age, weight, and years of residence in an endemic area; the Fisher’s exact test for gender and IVM rounds (categorical); and the Kruskal-Wallis test for the number of IVM rounds, MF counts, and the number of nodules and nodule locations. The Wilcoxon signed-rank test was used for comparisons of MF counts from individual participants across time.

Alternating regression (GENMOD, SAS) was used to analyze the histological data. No covariables were included as effectors in the primary analyses.

Multivariable analyses were done using Proc Genmod for the histological variables and Proc Log for the analysis of microfiladermia at 18 and 36 months. The following covariables were included in the multivariable analyses: MF average at baseline, age, gender, years in endemic area, number of previous IVM rounds, previous MDA (yes/no), treatment with ALB in addition to IVM, and treatment interval (annual/semiannual).

## RESULTS

### Baseline Data

We randomly assigned 294 volunteers to 1 of the 4 treatment arms without statistically significant differences regarding the baseline parameters (Table 1). This included the median MF group densities, which were not statistically different (*P* = .588), despite the great range of maximum MF skin snip counts. There were 272 participants who received the study drugs at least once, in line with the initially planned sample size (Supplementary Table 1; Supplementary Figure 1). Of 272 participants, 218 (80%) got their onchocercomata surgically removed after 36 months. Of these, 197 (72.4%) had followed treatment according to protocol (PP analysis set). The drop-out rate of 27.6% corresponded to the originally expected drop-out rate of 30%.

**Table 1.** Baseline Data

		IVM annual	IVM semiannual	IVM + ALB annual	IVM + ALB semiannual	*P* Value
n		73	74	74	73	
**Gender**	Female	28 (38.4%)	27 (36.5%)	19 (25.7%)	26 (35.6%)	.351^a^
	Male	45 (61.6%)	47 (63.5%)	55 (74.3%)	47 (64.4%)	
**Age**	Mean ± SD	42.7 ± 9.3	39.7 ± 10.3	41.3 ± 11.3	40.5 ± 10.5	.337^b^
	95% CI of the mean	(40.5–44.9)	(37.3–42.1)	(38.7–43.9)	(38.1–43.0)	
	Min–Max	24–60	18–60	19–60	20–60	
**Years in endemic area**	Mean ± SD	29.3 ± 12.0	24.7 ± 10.0	26.3 ± 13.6	24.9 ± 12.7	.082^b^
	95% CI of the mean	(26.5–32.1)	(22.3–27.0)	(23.1–29.4)	(22.0–27.9)	
	Min–Max	6–60	3–45	4–55	5–60	
**Weight**	Mean ± SD	56.0 ± 9.0	59.1 ± 8.3	58.1 ± 8.5	57.1 ± 8.7	.154^b^
	95% CI of the mean	(53.9–58.1)	(57.2–61.1)	(56.2–60.1)	(55.1–59.2)	
	Min–Max	41–99	43–77	41–86	42–91	
**Number of previous IVM rounds**	0 rounds, n (%) [95% CI]^c^	21 (28.8%) [19.7–40]	22 (29.7%) [20.5–40.9]	21 (28.4%) [19.4–39.5]	21 (28.8%) [19.7–40]	1.0^a^
	1 round, n (%)	13 (17.8%)	12 (16.2%)	15 (20.3%)	20 (27.4%)	
	2 rounds, n (%)	14 (19.2%)	11 (14.9%)	12 (16.2%)	12 (16.4%)	
	3 rounds, n (%)	13 (17.8%)	13 (17.6%)	8 (10.8%)	11 (15.1%)	
	>3 rounds, n (%)	12 (16.4%)	16 (21.6%)	18 (24.3%)	9 (12.3%)	
**Previous IVM rounds**	Median	2	2	2	1	.644^e^
	95% CI of the median^d^	(1–2)	(1–3)	(1–2)	(1–2)	
	25th; 75th percentiles	0; 3	0; 3	0; 3	0; 3	
	Min–Max	0–8	0–10	0–10	0–8	
**Number of nodule locations**	Median	2	2	2	2	.944^e^
	95% CI of the median^d^	(2–2)	(1–2)	(1–2)	(1–2)	
	25th; 75th percentiles	1; 2	1; 2	1; 2	1; 2	
	Min–Max	1–5	1–4	1–5	1–5	
**Number of nodules**	Median	2	2	3	2	.964^e^
	95% CI of the median^d^	(2–3)	(2–3)	(2–3)	(2–3)	
	25th; 75th percentiles	2; 4	1; 3	1; 4	2; 4	
	Min–Max	1–9	1–12	1–7	1–13	
**MF/mg skin**	Median	3.6	3.3	5.2	2.7	.588^e^
	95% CI of the median^d^	(2.6–7.1)	(1.4–5.6)	(3–7.7)	(1.7–5.7)	
	25th; 75th percentiles	1.1; 12.6	0.8; 17.1	1; 14.8	0.8; 9.9	
	Min–Max	0.2–69.5	0.1–113.4	0.1–367.3	0.1–158.6	

Abbreviations: ALB, albendazole; CI, confidence interval; IVM, ivermectin; MF, microfilaria; SD, standard deviation.

^a^Fisher’s exact test.

^b^Analysis of variance.

^c^CIs for proportions were calculated using the method recommended by Altman et al [30].

^d^CIs for the median were calculated using bootstrapping.

^e^Kruskal-Wallis test.

### Treatment and Follow-ups

**Figure 2.**
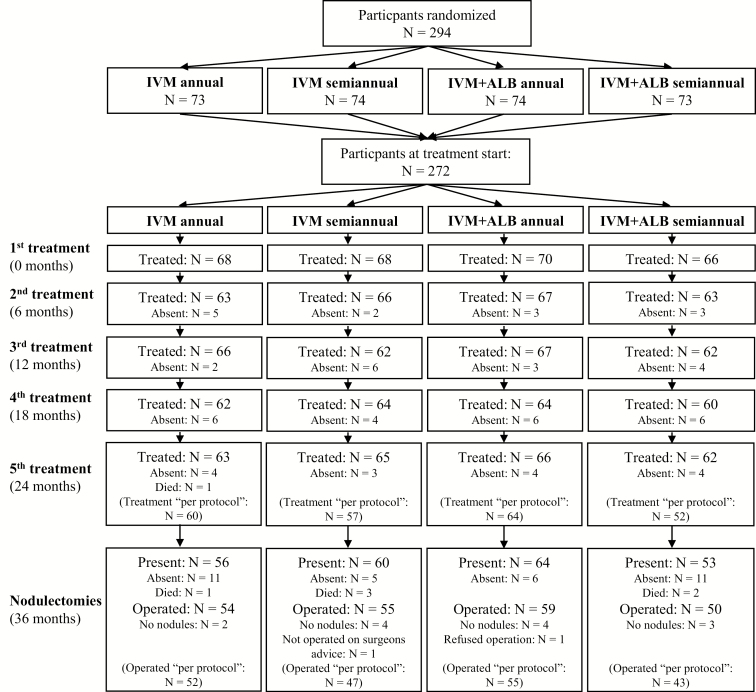
Participant flow chart, showing the number of participants randomized, treated, and operated. Participants who were absent for 1 or more treatments were always invited to continue treatment at the next visit or to come for the nodulectomies. Therefore, the number of absent participants changed between the respective visits. All participants that took part in the treatment or nodulectomies with no major violations to the protocol are listed in parentheses as “per protocol.” In total, 294 patients were randomized, but 22 participants did not take part in the treatment at all, due to pregnancy (n = 1), traveling (n = 10), refusal to participate (n = 3), moving (n = 3), and medical reasons (n = 5). To reach the initially planned number of 272 participants, 22 additional patients were consecutively randomly assigned. The first treatment (n = 272) was carried out in 2 batches: the first group (n = 182) was treated from 9–22 February 2013 and the second group (n = 90) from 6–13 April 2013. The second treatment (n = 259; 95.2%) was carried out 5.5 ± 0.6 months (range 5–8) after the first treatment. The third treatment (n = 257; 94.5%) was carried out 12 ± 0.8 months (range 11–13) after the first treatment and 5.9 ± 0.9 months (range 4–7) after the second treatment. The fourth treatment (n = 251; 92.3%) was carried out 17.8 ± 0.8 months (range 16–19) after the first treatment and 5.4 ± 0.5 months (range 4–6) after the third treatment. The fifth treatment (n = 254; 93.4%) was carried out 23.8 ± 0.8 months (range 22–25) after the first treatment and 5.7 ± 0.5 months (range 5–6) after the fourth treatment. The nodulectomies (n = 233; 85.7%) were carried 35.4 ± 0.9 months (range 34–36) after the first treatment and 10.9 ± 0.4 months (range 10–12) after the fifth treatment. Abbreviations: ALB, albendazole; IVM, ivermectin.

The study timeline is shown in Figure 1. Participation in treatment and follow-ups are described in Figure 2.

### Histological Analysis of the Onchocercomata

#### Live Versus Dead Female Worms

**Figure 3.**
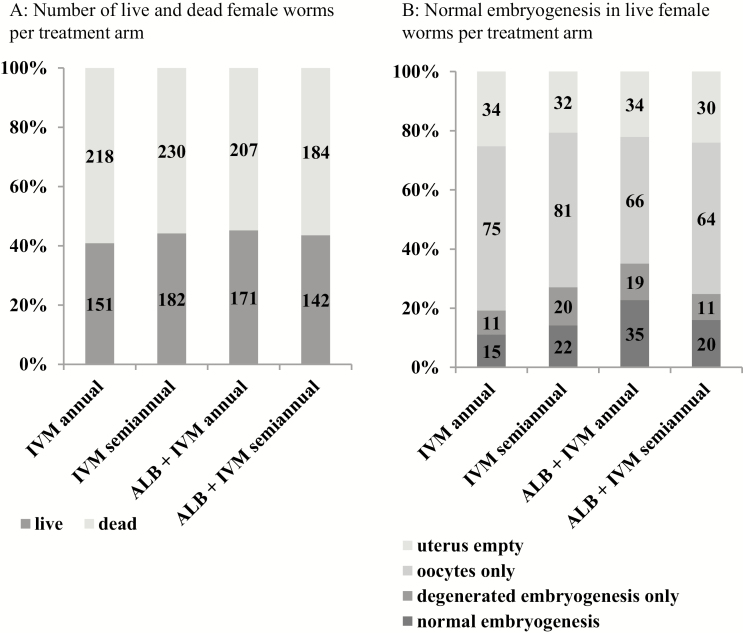
Histology (intention to treat). *A*, The number of live and dead female worms, per treatment arm. *B*, The embryogenesis in live female worms, per treatment arm. Abbreviations: ALB, albendazole; IVM, ivermectin.

With a range between 54.8–59.1%, the proportion of dead female worms did not differ between the treatment groups (ITT *P* = .9198; PP analysis *P* = .7206). This also applied when only patients without previous IVM treatment were considered (ITT range 48.4–65.8%; *P* = .5987) (Figure 3A; Supplementary Tables 2*A–D*).

The multivariable analysis did not reveal any effect on the variable live/dead female worms.

#### Normal Embryogenesis

As shown in Figure 3B, Table 2, and Supplementary Tables 3*A–C*, participants had 15/135 (11.1%), 22/155 (14.2%), 35/154 (22.7%), and 20/125 (16.0%) living female worms with normal embryogenesis in the IVM annual, IVM semiannual, IVM+ALB annual, and IVM+ALB semiannual groups, respectively (*P* = .1229). The PP analysis confirmed the ITT analysis (*P* = .1722). Equivalence was also observed when only patients without previous IVM treatment were considered (ITT *P* = .6877; PP *P* = .4134). The addition of ALB to IVM did not reduce female worm fertility. Indeed, recipients of combination treatment had slightly higher percentages of female worms with normal embryogenesis than recipients of IVM alone (ITT *P* = .0354, odds ratio 1.78 [95% confidence interval 1.04–3.06]; PP: *P* = .0526; odds ratio 1.73 [95% confidence interval 0.99–3.02]). Another measure of female fecundity is the presence of free MF in nodule tissue or insemination of live female worms (Supplementary Tables 4*A* and *B* and 5*A* and *B*). Neither of these 2 parameters showed differences between the 4 treatments (*P* = .3731 in free MF nodule tissue; *P* = .1179 in insemination).

**Table 2.** Embryogenesis: Intention to Treat

		Number of Living Female Worms					
			Embryogenesis				
Treatment	Number of Patients/Nodules	All	Judgement Not Possible^a^	Uterus Empty	Oocytes Only	Degenerated Embryogenesis Only	Normal Embryogenesis^b,c^
**IVM annual**	54/155	151	16	34	75	11 (8.1% [5–14])	15 (11.1% [7–18])
**IVM semiannual**	55/165	182	27	32	81	20 (12.9% [9–19])	22 (14.2% [10–21])
**IVM + ALB annual**	59/157	171	17	34	66	19 (12.3% [8–18])	35 (22.7% [17–30])
**IVM + ALB semiannual**	48/140	142^d^	17	30	63	11 (8.8% [5–15])	20 (16% [11–23])
All	216/617^e^	646	77	130	286	61 (10.7% [8; 14])	92 (16.2% [13; 19])

Data are given as n (% [95% confidence interval]).

Abbreviations: ALB, albendazole; IVM, ivermectin.

^a^In 77 out of the 646 live female worms, the judgement of the embryogenesis was not possible: for example, due to bad quality of the slide, the uterus not being truncated, indefinable contents of the uterus, or folded worm sections.

^b^95% confidence intervals for proportions were calculated using the recommended method by Altman et al [30].

^c^Comparison of all groups (live female worms with normal embryogenesis vs all other live female worms with evaluated embryogenesis): *P* = .1229 (Proc Genmod, SAS).

^d^In 1 live female worm, judgement of embryogenesis was possible but could not be assigned to 1 of the embyrogenic stages, as the worm was neoplastic. Therefore, the described stages sum up to 141 instead of 142. The worm has been analyzed as a worm with no normal embryogenesis.

^e^The nodules from 2 out of 218 patients could not be analyzed, because no oncho nodules or no worm section could be found (IVM + ALB semiannual n = 2); 60 nodules out of 677 nodules analyzed were not evaluable (IVM annual n = 8, IVM semiannual n = 14, IVM + ALB annual n = 22, IVM + ALB semiannual n = 16).

### Microfiladermia

**Figure 4.**
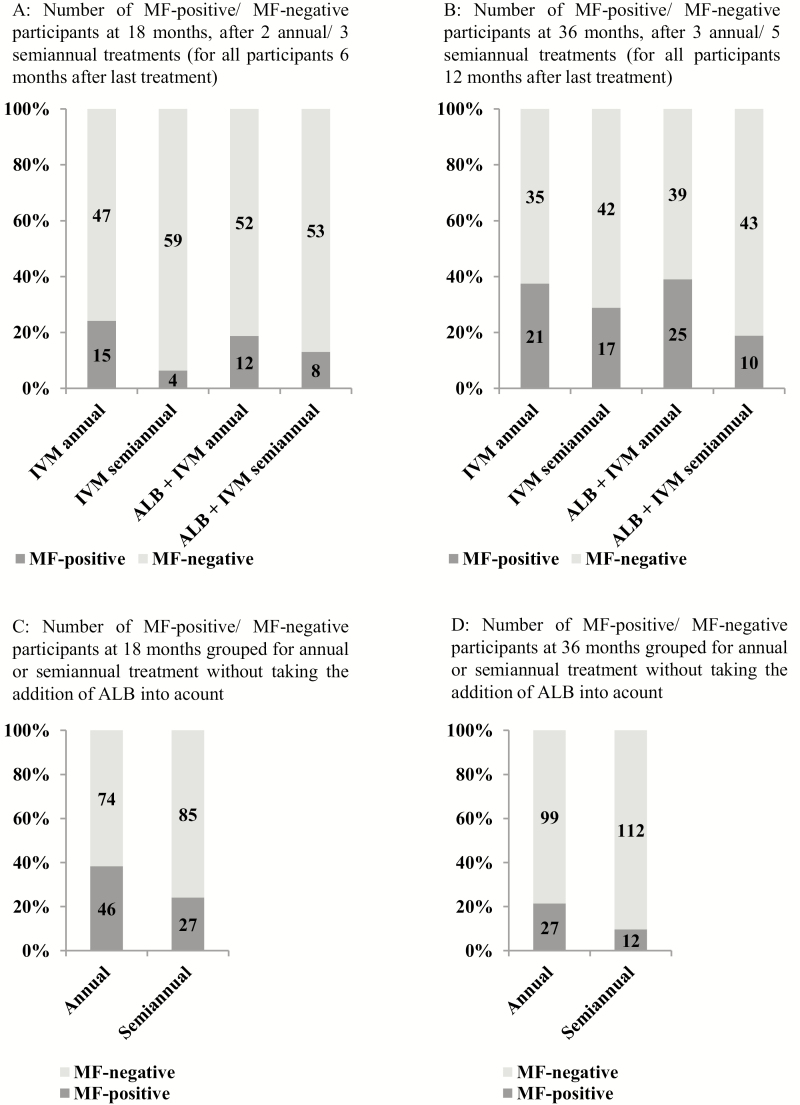
Absence of MF in the skin (intention to treat). *A*, The number of participants with/without MF at 18 months, after 2 annual/3 semiannual treatments (for all participants at 6 months after the last treatment). *B*, The number of participants with/without MF at 36 months, after 3 annual/5 semiannual treatments (for all participants at 12 months after the last treatment). *C*, The same participants as in panel *A*, at 18 months, but only grouped for annual or semiannual treatment, without taking the addition of ALB into account. *D*, The same participants as in panel *B*, at 36 months, but only grouped for annual or semiannual treatment, without taking the addition of ALB into account. Abbreviations: ALB, albendazole; IVM, ivermectin; MF, microfilaria.

A significant difference regarding the rates of individuals without MF among the 4 groups was detected at 18 months, with 75.8% in the IVM annual, 93.7% in the IVM semiannual, 81.2% in the IVM+ALB annual, and 86.9% in the IVM+ALB semiannual groups (Kruskal-Wallis, *P* = .035). A similar trend was present when we compared MF/mg skin among the 4 groups at this time point (*P* = .051). Comparing the annual and semiannual treatment (±ALB) groups, 21.4% in the annual group but only 9.7% in the semiannual group tested positive for MF at 18 months. A multivariable analysis clearly revealed that, beside the baseline counts of MF/mg skin (*P* < .001), the semiannual drug administration had a significant influence on the clearance of MF (*P* = .011). This result was confirmed by the PP analysis (Figures 4A–D; Tables 3 and 4; Supplementary Tables 6*A–F*).

**Table 3.** Microfiladermia: Intention to Treat

		IVM annual	IVM semiannual	IVM + ALB annual	IVM + ALB semiannual	*P* Value
Baseline	n	68	68	70	66	
MF positive	n (%)	68 (100%)	68 (100%)	70 (100%)	66 (100%)	
MF/mg skin	Median	4.5	3.3	5.2	3.6	
	95% CI of the median^a^	(2.9–7.6)	(1.4–5.7)	(2.9–8.1)	(1.7–6.2)	
	Geometric mean^b^	5.2	4.5	5.7	5.5	
	Min–Max	0.2–69.5	0.1–113	0.1–367	0.1–159	
	25th; 75th percentiles	1.2; 14.1	0.8; 15.2	0.9; 14.8	0.9; 10.8	.77^c^
6 months	n	63	66	67	63	
MF positive	n (%) [95%CI]^d^	26 (41.3%) [30–53.6]	26 (39.4%) [28.5–51.5]	30 (44.8%) [33.5–56.6]	18 (28.6%) [18.9–40.7]	.262^e^
MF/mg skin	Median	0	0	0	0	
	95% CI of the median^a^	(0–.25)	(0–.13)	(0–.29)	(0–0)	
	Geometric mean^b^	0.25	0.25	0.35	0.19	
	Min–Max	0–4.2	0–4.6	0–13.9	0–3.3	
	25th; 75th percentiles	0; 0.3	0; 0.3	0; 0.6	0; 0.2	.268^c^
*P* Value (comparison to baseline)^f^		*P* < .001	*P* < .001	*P* < .001	*P* < .001	
18 months	n	62	63	64	61	
MF positive	n (%) [95%CI]^d^	15 (24.2%) [15.2–36.2]	4 (6.3%) [2.5–15.2]	12 (18.8%) [11.1–30]	8 (13.1%) [6.8–23.8]	**.035** ^**e**^
MF/mg skin	Median	0	0	0	0	
	95% CI of the median^a^	(0–0)	(0–0)	(0–0)	(0–0)	
	Geometric mean^b^	0.09	0.02	0.11	0.06	
	Min–Max	0–3.7	0–0.7	0–4.2	0–1.6	
	25th; 75th percentiles	0; 0	0; 0	0; 0	0; 0	.051^c^
*P* Value (comparison to baseline)^f^		*P* < .001	*P* < .001	*P* < .001	*P* < .001	
36 months	n	56	59	64	53	
MF positive	n (%) [95%CI]^d^	21 (37.5%) [26–50.6]	17 (28.8%) [18.8–41.4]	25 (39.1%) [28.1–51.3]	10 (18.9%) [10.6–31.4]	.075^e^
MF/mg skin	Median	0	0	0	0	
	95% CI of the median^a^	(0–0.05)	(0–0)	(0–0.05)	(0–0)	
	Geometric mean^b^	0.2	0.15	0.23	0.16	
	Min–Max	0–6.3	0–5.6	0–11.1	0–6.1	
	25th; 75th percentiles	0; 0.3	0; 0.1	0; 0.2	0; 0	.139^c^
*P* Value (comparison to baseline)^f^		*P* < .001	*P* < .001	*P* < .001	*P* < .001	

These *P*-values are written in bold to show the significant values below .05.

Abbreviations: ALB, albendazole; CI, confidence interval; IVM, ivermectin; MF, microfilaria.

^a^CIs for the median were calculated using bootstrapping.

^b^The geometric mean was calculated by adding 1 to the original MF values and subtracting 1 from the final result.

^c^Kruskal-Wallis test.

^d^CIs for proportions were calculated using the method recommended by Altman et al [30].

^e^Fisher’s exact test.

^f^Wilcoxon signed-rank test.

**Table 4.** Microfiladermia: Intention to Treat, Annual Versus Semiannual Treatment

		Annual Treatment	Semiannual Treatment	*P* Value
Baseline	n	138	134	
MF positive	n (%)	138 (100%)	134 (100%)	
MF/mg skin	Median	4.9	3.4	
	95% CI of the median^a^	(3.4–7.1)	(2.1–5.4)	
	Geometric mean^b^	5.4	4.4	
	Min–Max	0.1–367	0.1–159	
	25th; 75th percentiles	1.1; 14.3	0.8; 11.1	.29^c^
6 months	n	130	129	
MF positive	n (%)	56 (43.1%)	44 (34.1%)	.161^d^
	95% CI^e^	(34.9–51.7)	(26.5–42.6)	
MF/mg skin	Median	0	0	
	95% CI of the median^a^	(0–0.2)	(0–0)	
	Geometric mean^b^	0.3	0.22	
	Min–Max	0–13.9	0–4.6	
	25th; 75th percentiles	0; 0.4	0; 0.3	.118^c^
	*P* value (comparison to baseline)^f^	*P* < .001	*P* < .001	
18 months	n	126	124	
MF positive	n (%)	27 (21.4%)	12 (9.7%)	**.014** ^**d**^
	95% CI^e^	(15.2–29.4)	(5.6–16.2)	
MF/mg skin	Median	0	0	
	95% CI of the median^a^	(0–0)	(0–0)	
	Geometric mean^b^	0.1	0.04	
	Min–Max	0–4.2	0–1.6	
	25th; 75th percentiles	0; 0	0; 0	**.012** ^**c**^
	*P* value (comparison to baseline)^f^	*P* < .001	*P* < .001	
36 months	n	120	112	
MF positive	n (%)	46 (38.3%)	27 (24.1%)	**.024** ^**d**^
	95% CI^e^	(30.1–47.3)	(17.1–32.8)	
MF/mg skin	Median	0	0	
	95% CI of the median^a^	(0–0)	(0–0)	
	Geometric mean^b^	0.2	0.15	
	Min–Max	0–11.1	0–6.1	
	25th; 75th percentiles	0; 0.2	0; 0	**.03** ^**c**^
	*P* value (comparison to baseline)^f^	*P* < .001	*P* < .001	

These *P*-values are written in bold to show the significant values below .05.

Abbreviations: ALB, albendazole; CI, confidence interval; IVM, ivermectin; MF, microfilaria.

^a^CIs for the median were calculated using bootstrapping.

^b^The geometric mean was calculated by adding 1 to the original MF values and subtracting 1 from the final result.

^c^Kruskal-Wallis test.

^d^Fisher’s exact test.

^e^CIs for proportions were calculated using the method recommended by Altman et al [30].

^f^Wilcoxon signed-rank test.

The proportion of individuals that completely cleared MF at 36 months were 63% in the IVM annual, 71% in the IVM semiannual, 61% in the IVM+ALB annual, and 81% in the IVM+ALB semiannual group. The addition of ALB did not improve or sustain MF clearance. However, the semiannual drug administration resulted in superior sustained MF clearance (annual 62%; semiannual 76%; *P* = .024). This result was confirmed by the multivariable analysis, where, in addition to the baseline MF/mg skin counts (*P* = .011), semiannual drug administration was significantly associated with the sustained clearance of MF (*P* = .029). A similar trend was seen in the PP analysis (MF/mg skin at baseline *P* = .006; annual/semiannual *P* = .064).

### Adverse Events

A total of 617 adverse events were reported during the 5 treatment rounds; 415 (67.3%) of these occurred after the first treatment, with no differences between the 4 treatment groups. The adverse events included cutaneous itching (31.4%), different types of pain (14.1%), swollen limbs (10.4%), headache (8.8%), cutaneous rash (6.3%), fever (6%), swollen face (2%), and other conditions, such as nausea, dizziness, vomiting, abdominal discomfort, and ocular reactions (n = 7; blurred vision ± itching painful eyes, all resolved without any residues). During the conduct of the study, 6 study participants died. None of the deaths were related to the study drugs (IVM annual n = 1, IVM semiannual n = 3; IVM+ALB semiannual n = 2) and all deaths happened 7–10 months after the patients received their last verum treatment. No other serious adverse events were observed (Table 5; Supplementary Table 7).

**Table 5.** Adverse Events: Number of Patients With Adverse Events/Total Number of Patients

	IVM annual	IVM semiannual	IVM + ALB annual	IVM + ALB semiannual
0 months	43/68 (63.2%)	48/67 (71.6%)	50/70 (71.4%)	45/66 (68.2%)
6 months	11/62 (17.7%)	16/66 (24.2%)	5/66 (7.6%)	16/63 (25.4%)
12 months	9/66 (13.6%)	6/62 (9.7%)	13/67 (19.4%)	11/62 (17.7%)
18 months	6/62 (9.7%)	13/64 (20.3%)	4/64 (6.3%)	7/60 (11.7%)
24 months	8/63 (12.7%)	6/65 (9.2%)	12/66 (18.2%)	7/60 (11.7%)

Abbreviations: ALB, albendazole; IVM, ivermectin.

## DISCUSSION

The question of whether IVM plus ALB is superior to IVM alone for onchocerciasis was raised 25 years ago, when Awadzi and Buettner examined the efficacy of a single dose of this combination in onchocerciasis patients [11, 19, 20]. The rationale for their study was that, while IVM preferentially acts on late embryonic stages in the female worm uterus (pretzel stages and stretched MF), ALB has embryotoxic effects [12] that manifest as 66% suppression of MF counts for at least 1 year [11]. However, administration of a single, 400 mg dose of ALB, combined with IVM at 200 µg/kg, failed to a show greater reduction in MF, as compared to IVM alone [19, 20]. These studies involved small numbers of participants, used ALB only at a dose of 400 mg, and followed the participants for just 1 year. In the present trial, we wanted to find out whether IVM combined with ALB at higher doses (800 mg), given multiple times, and given up to twice per year for 3 years might generate a more sustained reduction in MF or adult female worms. This is the first RCT that has dealt with this question in a patient cohort where the sample size calculation was done such that it would have also picked up a difference in adult female worm fertility of only 15% (see Supplementary Figure 1). Our data show that neither the viability nor fertility of female worms were significantly reduced by the addition of ALB. The addition of ALB also did not enhance MF clearance at the 18- or 36-month follow-up time points. Instead, our study confirmed earlier studies [21] that suggested that switching from annual to semiannual treatment with IVM, with or without ALB, would result in significantly lower MF burdens. Notably, this was also observed here at 36 months (Table 4), 1 year after the last treatment. MF in the skin at that time reflect renewed production and release by female adult worms. This suggests that the more frequent administration of IVM results in reduced female worm fertility for a period of up to 1 year by a mechanism that might not be observed by histology.

The overall low proportion of adult female worms with ongoing embryo production (11.1–22.7%) is a limitation of this study. In historical ivermectin-naive hyperendemic areas, the expected proportion of dead adult female worms does not exceed approximately 20% [22, 23], and higher proportions are indicative of transmission reduction, with less young worms developing. Of the live female worms in these prior studies, approximately 30–40% did not produce embryos and presented with empty uteri or oocytes only [22]. These proportions were clearly exceeded in the current trial. Thus, the low number of live worms, the small size of nodules, and the fact that the majority of live worms were old (645 old worms/845 live worms; 77.3%) suggest that transmission has been low in the study area for some time. In addition, the subgroup of patients without previous IVM was only 27.9%. However, a multivariate analysis did not reveal that previous IVM affected the results.

Our results support the earlier smaller studies by Buettner and Awadzi [19, 20]; therefore, they are not too surprising. However, the data from this study (more participants, multiple treatment doses, a higher ALB dose, and longer follow-up) significantly expand on the prior studies. Another RCT with a very similar design was undertaken in an area with higher infection intensities, less prior ivermectin treatment, and potentially more young worms (NCT02078024); it will be interesting whether results from that trial confirm the current findings. Future studies planned within the DOLF consortium will also address whether moxidectin will add a cost or time-to-elimination advantage over IVM.

Our results support the idea of providing semiannual MDA with IVM, because it is more effective for suppressing MF and because it may have a cumulative effect on adult worm fertility. This issue has been widely debated in the literature [24–27], and it seems the consensus is that biannual treatment does not dramatically improve health gains, but would reduce the time to elimination drastically, saving billions of US dollars [7]. Our study has provided new data on this issue that may be useful for modelling studies [5]. Our results do not suggest that ALB plus IVM is superior to IVM alone for the treatment of onchocerciasis. However, the impetus for adding ALB to IVM came from LF elimination programs, in response to frequent co-endemicity of onchocerciasis with LF. The Global Programme to Eliminate LF has long recommended the addition of ALB to IVM for LF elimination in areas of Africa, based on results from clinical trials [28, 29]. Although our study did not find that IVM plus ALB had increased activity against *O. volvulus*, we could also argue that the combination should be used for onchocerciasis elimination even in areas without LF, because of the STH benefit that ALB provides. The STH benefit is considerable, and it may improve compliance with MDA in some settings.

## Supplementary Data

Supplementary materials are available at *Clinical Infectious Diseases* online. Consisting of data provided by the authors to benefit the reader, the posted materials are not copyedited and are the sole responsibility of the authors, so questions or comments should be addressed to the corresponding author.

ciz889_suppl_Supplementary_Tables

## Notes

***Acknowledgments.*** The authors thank Dr Nana-Kwadwo Biritwum, Programme Manager for NTDs at the Ghana Ministry of Health, for providing us with the study drugs (ivermectin); the Adanse North and South District health directorate for their support in selecting study sites for the conduct of the study; the disease control officers of the districts and the community health volunteers of the various communities for their involvement in the organization of patients; and FHI360 (Family Health International) for monitoring the study.

***Disclaimer.*** The content of this publication is solely the responsibility of the authors and does not represent the official views of the Bill & Melinda Gates Foundation.

***Financial support.*** This work was supported by the Bill & Melinda Gates Foundation (grant number OPPGH5342 to The Death to Onchocerciasis and Lymphatic Filariasis Project).

***Potential conflicts of interest.*** The authors report no potential conflicts of interest. All authors have submitted the ICMJE Form for Disclosure of Potential Conflicts of Interest. Conflicts that the editors consider relevant to the content of the manuscript have been disclosed.

## References

[1] Simonsen PE, Fischer PU, Hoerauf A, Weil G. In: Farrar J, Hotez P, Junghanss T, Kang G, Lalloo D, White N. eds. Chapter: The filariases. Book: Manson’s tropical infectious diseases. 23rd ed. Edinburgh, UK: Elsevier, Saunders Ltd., 2014: 737–65.e5.

[2] Basáñez MG, Pion SD, Churcher TS, Breitling LP, Little MP, Boussinesq M River blindness: a success story under threat? PLOS Med 2006; 3:e371. 17002504 10.1371/journal.pmed.0030371 PMC1576321

[3] Hopkins AD Neglected tropical diseases in Africa: a new paradigm. Int Health 2016; 8(Suppl 1):i28–33. 26940307 10.1093/inthealth/ihv077

[4] Basáñez MG, Pion SD, Boakes E, Filipe JA, Churcher TS, Boussinesq M Effect of single-dose ivermectin on *Onchocerca volvulus*: a systematic review and meta-analysis. Lancet Infect Dis 2008; 8:310–22. 18471776 10.1016/S1473-3099(08)70099-9

[5] Walker M, Stolk WA, Dixon MA, et al. Modelling the elimination of river blindness using long-term epidemiological and programmatic data from Mali and Senegal. Epidemics 2017; 18:4–15. 28279455 10.1016/j.epidem.2017.02.005 PMC5340858

[6] World Health Organization. Report of the consultative meetings on strategic options and alternative treatment strategies for accelerating onchocerciasis elimination in Africa. Geneva, Switzerland: World Health Organization, 2015.

[7] Kim YE, Sicuri E, Tediosi F Financial and economic costs of the elimination and eradication of onchocerciasis (river blindness) in Africa. PLOS Negl Trop Dis 2015; 9:e0004056. 26360917 10.1371/journal.pntd.0004056 PMC4567329

[8] Awadzi K, Opoku NO, Attah SK, Lazdins-Helds J, Kuesel AC A randomized, single-ascending-dose, ivermectin-controlled, double-blind study of moxidectin in *Onchocerca volvulus* infection. PLOS Negl Trop Dis 2014; 8:e2953. 24968000 10.1371/journal.pntd.0002953 PMC4072596

[9] Turner HC, Walker M, Attah SK, et al. The potential impact of moxidectin on onchocerciasis elimination in Africa: an economic evaluation based on the Phase II clinical trial data. Parasit Vectors 2015; 8:167. 25889256 10.1186/s13071-015-0779-4 PMC4381491

[10] Opoku NO, Bakajika DK, Kanza EM, et al. Single dose moxidectin versus ivermectin for *Onchocerca volvulus* infection in Ghana, Liberia, and the Democratic Republic of the Congo: a randomised, controlled, double-blind phase 3 trial. Lancet 2018; 392:1207–16. 29361335 10.1016/S0140-6736(17)32844-1 PMC6172290

[11] Awadzi K, Hero M, Opoku O, Büttner DW, Gilles HM The chemotherapy of onchocerciasis. XV. Studies with albendazole. Trop Med Parasitol 1991; 42:356–60. 1796233

[12] Zahner H, Schares G Experimental chemotherapy of filariasis: comparative evaluation of the efficacy of filaricidal compounds in *Mastomys coucha* infected with *Litomosoides carinii*, *Acanthocheilonema viteae*, *Brugia malayi* and *B. pahangi*. Acta Trop 1993; 52:221–66. 8094587 10.1016/0001-706x(93)90010-9

[13] Hoerauf A, Specht S, Büttner M, et al. *Wolbachia endobacteria* depletion by doxycycline as antifilarial therapy has macrofilaricidal activity in onchocerciasis: a randomized placebo-controlled study. Med Microbiol Immunol 2008; 197:295–311. 17999080 10.1007/s00430-007-0062-1 PMC2668626

[14] Mand S, Marfo-Debrekyei Y, Debrah A, et al Frequent detection of worm movements in onchocercal nodules by ultrasonography. Filaria J 2005; 4:1. 15788103 10.1186/1475-2883-4-1 PMC1079913

[15] Specht S, Brattig N, Büttner M, Büttner DW Criteria for the differentiation between young and old *Onchocerca volvulus* filariae. Parasitol Res 2009; 105:1531–8. 19784672 10.1007/s00436-009-1588-5 PMC2764059

[16] Specht S, Hoerauf A, Adjei O, Debrah A, Büttner DW Newly acquired *Onchocerca volvulus* filariae after doxycycline treatment. Parasitol Res 2009; 106:23–31. 19756742 10.1007/s00436-009-1624-5 PMC2780640

[17] Klarmann-Schulz U, Specht S, Debrah AY, et al. Comparison of doxycycline, minocycline, doxycycline plus albendazole and albendazole alone in their efficacy against onchocerciasis in a randomized, open-label, pilot trial. PLOS Negl Trop Dis 2017; 11:e0005156. 28056021 10.1371/journal.pntd.0005156 PMC5215804

[18] Harris PA, Taylor R, Thielke R, Payne J, Gonzalez N, Conde JG Research electronic data capture (REDCap)–a metadata-driven methodology and workflow process for providing translational research informatics support. J Biomed Inform 2009; 42:377–81. 18929686 10.1016/j.jbi.2008.08.010 PMC2700030

[19] Awadzi K, Edwards G, Duke BO, et al. The co-administration of ivermectin and albendazole–safety, pharmacokinetics and efficacy against *Onchocerca volvulus*. Ann Trop Med Parasitol 2003; 97:165–78. 12803872 10.1179/000349803235001697

[20] Awadzi K, Addy ET, Opoku NO, Plenge-Bönig A, Büttner DW The chemotherapy of onchocerciasis XX: ivermectin in combination with albendazole. Trop Med Parasitol 1995; 46:213–20. 8826100

[21] Coffeng LE, Stolk WA, Hoerauf A, et al. Elimination of African onchocerciasis: modeling the impact of increasing the frequency of ivermectin mass treatment. PLOS One 2014; 9:e115886. 25545677 10.1371/journal.pone.0115886 PMC4278850

[22] Hoerauf A, Mand S, Volkmann L, et al. Doxycycline in the treatment of human onchocerciasis: Kinetics of *Wolbachia endobacteria* reduction and of inhibition of embryogenesis in female *Onchocerca* worms. Microbes Infect 2003; 5:261–73. 12706439 10.1016/s1286-4579(03)00026-1

[23] Hoerauf A, Specht S, Marfo-Debrekyei Y, et al. Efficacy of 5-week doxycycline treatment on adult *Onchocerca volvulus*. Parasitol Res 2009; 104:437–47. 18850111 10.1007/s00436-008-1217-8

[24] Churcher TS, Pion SD, Osei-Atweneboana MY, et al. Identifying sub-optimal responses to ivermectin in the treatment of river blindness. Proc Natl Acad Sci USA 2009; 106:16716–21. 19805362 10.1073/pnas.0906176106 PMC2757820

[25] Katabarwa M, Richards F Twice-yearly ivermectin for onchocerciasis: the time is now. Lancet Infect Dis 2014; 14:373–4. 24758997 10.1016/S1473-3099(14)70732-7

[26] Turner HC, Walker M, Churcher TS, et al. Reaching the London declaration on neglected tropical diseases goals for onchocerciasis: an economic evaluation of increasing the frequency of ivermectin treatment in Africa. Clin Infect Dis 2014; 59:923–32. 24944228 10.1093/cid/ciu467 PMC4166981

[27] Burki T River blindness elimination in Columbia. Lancet Infect Dis 2013; 13:922–3. 24319775 10.1016/s1473-3099(13)70308-6

[28] Ismail MM, Jayakody RL, Weil GJ, et al. Efficacy of single dose combinations of albendazole, ivermectin and diethylcarbamazine for the treatment of bancroftian filariasis. Trans R Soc Trop Med Hyg 1998; 92:94–7. 9692166 10.1016/s0035-9203(98)90972-5

[29] Ismail MM, Jayakody RL, Weil GJ, et al. Long-term efficacy of single-dose combinations of albendazole, ivermectin and diethylcarbamazine for the treatment of bancroftian filariasis. Trans R Soc Trop Med Hyg 2001; 95:332–5. 11491010 10.1016/s0035-9203(01)90257-3

[30] Altman DG, Machin D, Bryant TN, Gardner MJ. Statistics with confidence, Confidence intervals and statistical guidelines. 2nd ed. London, UK: British Medical Journal Books, 2000.

