# Supplementary material for: Comparison of Repeated Doses of Ivermectin Versus Ivermectin Plus Albendazole for the Treatment of Onchocerciasis: A Randomized, Open-label, Clinical Trial
Source: Clin Infect Dis. 2019 Sep 19;71(4):933–43. doi: 10.1093/cid/ciz889 (PMC7428389; doi:10.1093/cid/ciz889)
Supplement: ciz889_suppl_Supplementary_Tables [file ciz889_suppl_supplementary_tables.docx]

**Supplementary figures and tables**

*Supplementary figure 1: Closed testing procedure*

H_{p2=p4}_

H_{p1=p2=p3=p4}_

H_{p1=p2=p3}_

H_{p1=p2=p4}_

H_{p1=p3=p4}_

H_{p2=p3=p4}_

H_{p1=p2}_

H_{p1=p3}_

H_{p1=p4}_

H_{p2=p3}_

H_{p3=p4}_

H_{p1=p2∩p3=p4}_

H_{p1=p3∩p2=p4}_

H_{p1=p4∩p2=p3}_

*Legend for supplementary figure 1:*

*Power calculation/ Sample size: This study is a randomized controlled trial to compare four different treatment regimes concerning the reduction of the number of fertile female worms in the treated patients. If p_1_, p_2_, p_3_, and p_4_ are the rates of fertile female worms under the four treatments, following the closed testing procedure, all possible intersection hypotheses of the 6 elementary hypotheses p_i_ = p_j_ (i≠j ) will be tested with a two-sided regression analysis. Hypotheses of the type H_{p1=p2=p3=p4}_  and H_{p1=p2=p3}_ will be tested at a level of α=5%. Hypotheses of the type H_{p1=p2∩p3=p4}_
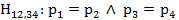

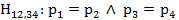
will be tested by testing H_{p1=p2}_ and H_{p3=p4}_ both at a level of α=2.5% and H_{p1=p2∩p3=p4}_ will be rejected if one of the two hypotheses had been rejected. Alternating logistic regression analysis (GENMOD, SAS®) is chosen for these tests, because it allows correcting for the possible dependency of the observation on different worms in one patient. To control the family wise error rate of all these tests an elementary hypothesis will only be rejected if all possible intersection hypotheses involving it have been rejected.* *To estimate the power for the intended approach, a simulation analysis was performed, assuming a dependency between the fertility rates of different female worms in the same person as estimated from available data. Assuming 1.5 worms per nodule and 3 nodules per person (4.5 worms per person) as well as fertility rates of 30% IVM annual and 20%, 15% and 10% for the experimental treatments of IVM semiannual, IVM + ALB annual and IVM + ALB semiannual, we found a power of 97% to detect a difference between the worst and the best and a power of 80% for the difference between the worst and the second best treatment regimen when choosing 52 participants per treatment group. Previous studies revealed a 20% loss to follow up 20 months after treatment, therefore a drop-out rate of 30% was calculated for this study with the last observation 36 months after treatment, resulting in 68 participants for each treatment group.*

*Supplementary table 1. Baseline data (treated)*

|  | | | **IVM**  **annual** | **IVM semiannual** | **IVM + ALB annual** | **IVM + ALB semiannual** | ***p-value*** |
| --- | --- | --- | --- | --- | --- | --- | --- |
|  | N | | 68 | 68 | 70 | 66 |  |
| **Gender** | female | | 28 (41.2%) | 24 (35.3%) | 17 (24.3%) | 23 (34.8%) | p = 0.198^c^ |
|  | male | | 40 (58.8%) | 44 (64.7%) | 53 (75.7%) | 43 (65.2%) |  |
| **Age (years)** | Mean ± SD | | 43.2 ± 9.1 | 40.0 ± 10.2 | 41.1 ± 11.1 | 40.9 ± 10.3 | p = 0.301^d^ |
|  | 95% CI of the mean | | [41.0; 45.4] | [37.5; 42.4] | [38.4; 43.7] | [38.4; 43.4] |  |
|  | Min - Max | | 24 - 60 | 18 - 60 | 19 - 60 | 20 - 60 |  |
| **Years in endemic area** | Mean ± SD | | 30.3 ± 11.7 | 24.5 ± 10.4 | 26.1 ± 12.9 | 24.8 ± 12.1 | ***p = 0.016^d,e^*** |
|  | 95% CI of the mean | | [27.4; 33.1] | [21.9; 27.0] | [23.0; 29.1] | [21.8; 27.7] |  |
|  | Min - Max | | 6 - 60 | 3 - 45 | 4 - 53 | 5 - 50 |  |
| **Weight** | Mean ± SD | | 55.8 ± 9.3 | 59.0 ± 8.3 | 58.0 ± 7.9 | 57.4 ± 8.9 | p = 0.181^d^ |
|  | 95% CI of the mean | | [53.6; 58.1] | [57.0; 61.0] | [56.1; 59.9] | [55.2; 59.6] |  |
|  | Min - Max | | 41 - 99 | 43 - 77 | 41 - 81 | 42 - 91 |  |
| **No. of previous IVM rounds** | **0** | N (%)  [95% CI]^a^ | 19 (27.9%)  [18.7; 39.6] | 22 (32.4%)  [22.4; 44.2] | 18 (25.7%)  [16.9; 37] | 17 (25.8%)  [16.7; 37.4] | p = 0.81^c^ |
|  | **1** | N (%) | 13 (19.1%) | 10 (14.7%) | 15 (21.4%) | 19 (28.8%) |  |
|  | **2** | N (%) | 12 (17.6%) | 11 (16.2%) | 12 (17.1%) | 11 (16.7%) |  |
|  | **3** | N (%) | 13 (19.1%) | 10 (14.7%) | 8 (11.4%) | 10 (15.2%) |  |
|  | **>3** | N (%) | 11 (16.2%) | 15 (22.1%) | 17 (24.3%) | 9 (13.6%) |  |
| **Previous IVM rounds** | Median | | 2 | 2 | 2 | 1 | p = 0.82^f^ |
|  | 95% CI of the median^b^ | | [1; 2] | [1; 2] | [1; 2] | [1; 2] |  |
|  | 25^th^; 75^th^ percentiles | | 0; 3 | 0; 3 | 0; 3 | 0; 3 |  |
|  | Min - Max | | 0 - 8 | 0 - 10 | 0 - 10 | 0 - 8 |  |
| **No. of nodule locations** | Median | | 2 | 2 | 2 | 2 | p = 0.949^f^ |
|  | 95% CI of the median^b^ | | [2; 2] | [1; 2] | [1; 2] | [1; 2] |  |
|  | 25^th^; 75^th^ percentiles | | 1; 2 | 1; 2 | 1; 3 | 1; 2 |  |
|  | Min - Max | | 1 - 5 | 1 - 4 | 1 - 5 | 1 - 5 |  |
| **No. of nodules** | Median | | 2 | 2 | 3 | 2 | p = 0.979^f^ |
|  | 95% CI of the median^b^ | | [2; 3] | [2; 3] | [2; 3] | [2; 3] |  |
|  | 25^th^; 75^th^ percentiles | | 2; 4 | 1; 3.5 | 1; 4 | 2; 4 |  |
|  | Min - Max | | 1 - 9 | 1 - 12 | 1 - 7 | 1 - 13 |  |
| **MF/mg skin** | Median | | 4.5 | 3.3 | 5.2 | 3.6 | p = 0.77^f^ |
|  | 95% CI of the median^b^ | | [2.9; 7.4] | [1.4; 5.7] | [2.9; 7.9] | [1.7; 6.2] |  |
|  | 25^th^; 75^th^ percentiles | | 1.2; 14.1 | 0.8; 15.2 | 0.9; 14.8 | 0.9; 10.8 |  |
|  | Min - Max | | 0.2 - 69.5 | 0.1 - 113.4 | 0.1 - 367.3 | 0.1 - 158.6 |  |

^a^ Confidence intervals for proportions were calculated using the recommended method by Altman et al. [28]

^b^ Confidence intervals for the median were calculated using bootstrapping

^c^ Fisher’s exact test

^d^ ANOVA

^e^ Significant difference between IVM annual and IVM semiannual (*p = 0.023*) and between IVM annual and IVM+ALB semiannual (*p = 0.038*, Tukey HSD posthoc test) meaning that IVM annual participants lived longer in an endemic area than semiannual treated participants. This fact did not have any influence on the results described in the paper (tested with multivariable analyses).

^f^ Kruskal-Wallis-test

*Supplementary table 2a. Live/dead worms (ITT)*

| Treatment | No. of  patients/  nodules | Total no. of worms | No. of female worms | | | No. of male worms | | |
| --- | --- | --- | --- | --- | --- | --- | --- | --- |
|  |  |  | All | Live | Dead^a,b^ | All | Live | Dead^a,c^ |
| **IVM**  **annual** | 54/155 | 427 | 369 | 151 (40.9%) | 218  (59.1% [54; 64]) | 58 | 50 (86.2%) | 8  (13.8% [7; 25]) |
| **IVM semiannual** | 55/165 | 466 | 412 | 182 (44.2%) | 230  (55.8% [51; 61]) | 54 | 47  (87%) | 7  (13% [6; 24]) |
| **IVM + ALB annual** | 59/157 | 438 | 378 | 171 (45.2%) | 207  (54.8% [50; 60]) | 60 | 50 (83.3%) | 10  (16.7% [9; 28]) |
| **IVM + ALB semiannual** | 48/140 | 376 | 326 | 142 (43.6%) | 184  (56.4% [51; 62]) | 50 | 41  (82%) | 9  (18% [10; 31]) |
| All | 216/617* | 1707 | 1485 | 646 (43.5%) | 839  (56.5% [50; 55]) | 222 | 188 (84.7%) | 34  (15.3% [11; 21]) |

* The nodules from 2 out of 218 patients could not be analysed because no oncho nodules or no worm section could be found (IVM + ALB semiannual N = 2), 60 nodules out of 677 nodules analysed were not evaluable (IVM annual N = 8, IVM semiannual N = 14, IVM + ALB annual N = 22, IVM + ALB semiannual N = 16)

^a^ 95% confidence intervals for proportions were calculated using the recommended method by Altman et al. [28]

^b^ Comparison of all groups (live female worms vs. dead female worms): p = 0.9198 (Proc Genmod, SAS®)

^c^ Comparison of all groups (live male worms vs. dead male worms): p = 0.8731 (Proc Genmod, SAS®)

*Supplementary table 2b. Live/dead worms (PP)*

| Treatment | No. of  patients/  nodules | Total no. of worms | No. of female worms | | | No. of male worms | | |
| --- | --- | --- | --- | --- | --- | --- | --- | --- |
|  |  |  | All | Live | Dead^a,b^ | All | Live | Dead^a,c^ |
| **IVM annual** | 52/152 | 419 | 363 | 147 (40.5%) | 216  (59.5% [54; 64]) | 56 | 48  (85.7%) | 8  (14.3% [7; 26]) |
| **IVM semiannual** | 47/142 | 397 | 349 | 161 (46.1%) | 188  (53.9% [49; 59]) | 48 | 43  (89.6%) | 5  (10.4% [5; 22]) |
| **IVM + ALB annual** | 55/141 | 399 | 347 | 160 (46.1%) | 187  (53.9% [49; 59]) | 52 | 46  (88.5%) | 6  (11.5% [5; 23]) |
| **IVM + ALB semiannual** | 42/128 | 352 | 303 | 127 (41.9%) | 176  (58.1% [52; 64]) | 49 | 40  (81.6%) | 9  (18.4% [10; 31]) |
| All | 196/563* | 1567 | 1362 | 595 (43.7%) | 767  (56.3% [54; 59]) | 205 | 177 (86.3%) | 28  (13.7% [10; 19]) |

*The nodules from 1 patient out of 197 could not be analysed because no oncho nodules or no worm section could be found (IVM + ALB semiannual N = 1), 52 nodules out of 615 nodules analysed were not evaluable (IVM annual N = 8, IVM semiannual N = 9, IVM + ALB annual N = 21, IVM + ALB semiannual N = 14)

^a^ 95% confidence intervals for proportions were calculated using the recommended method by Altman et al. [28]

^b^ Comparison of all groups (live female worms vs. dead female worms): p = 0.7206 (Proc Genmod, SAS®)

^c^ Comparison of all groups (live male worms vs. dead male worms): p = 0.6943 (Proc Genmod, SAS®)

*Supplementary table 2c. Live/dead worms (ITT, no previous MDA)*

| Treatment | No. of patients/  nodules | Total no. of worms | No. of female worms | | | No. of male worms | | |
| --- | --- | --- | --- | --- | --- | --- | --- | --- |
|  |  |  | All | Live | Dead^a,b^ | All | Live | Dead^a,c^ |
| **IVM**  **annual** | 14/40 | 107 | 91 | 47  (51.6%) | 44  (48.4% [38; 58]) | 16 | 15 (93.8%) | 1  (6.3% [1; 28]) |
| **IVM semiannual** | 17/65 | 172 | 152 | 52  (34.2%) | 100  (65.8% [58; 73]) | 20 | 17  (85%) | 3  (15% [5; 36]) |
| **IVM + ALB annual** | 14/29 | 87 | 75 | 35  (46.7%) | 40  (53.3% [42; 64]) | 12 | 9  (75%) | 3  (25% [9; 53]) |
| **IVM + ALB semiannual** | 10/23 | 61 | 50 | 24  (48%) | 26  (52% [39; 65]) | 11 | 10 (90.9%) | 1  (9.1% [2; 38]) |
| All | 55/157 | 427 | 368 | 158 (42.9%) | 210  (57.1% [52; 62]) | 59 | 51 (86.4%) | 8  (13.6% [7; 25]) |

^a^ 95% confidence intervals for proportions were calculated using the recommended method by Altman et al. [28]

^b^ Comparison of all groups (live female worms vs. dead female worms): p = 0.5987 (Proc Genmod, SAS®)

^c^ Comparison of all groups (live male worms vs. dead male worms): p = 0.5163 (Proc Genmod, SAS®)

*Supplementary table 2d. Live/dead worms (PP, no previous MDA)*

| Treatment | No. of patients/  nodules | Total no. of worms | No. of female worms | | | No. of male worms | | |
| --- | --- | --- | --- | --- | --- | --- | --- | --- |
|  |  |  | All | Live | Dead^a,b^ | All | Live | Dead^a,c^ |
| **IVM**  **annual** | 13/38 | 103 | 88 | 44  (50%) | 44  (50% [40; 60]) | 15 | 14 (93.3%) | 1  (6.7% [1; 30]) |
| **IVM semiannual** | 15/60 | 153 | 136 | 49  (36%) | 87  (64% [56; 72]) | 17 | 15 (88.2%) | 2  (11.8% [3; 34]) |
| **IVM + ALB annual** | 11/22 | 70 | 62 | 32  (51.6%) | 30  (48.4% [36; 61]) | 8 | 7  (87.5%) | 1  (12.5% [2; 47]) |
| **IVM + ALB semiannual** | 6/18 | 50 | 40 | 16  (40%) | 24  (60% [45; 74]) | 10 | 9  (90%) | 1  (10% [2; 40]) |
| All | 45/138 | 376 | 326 | 141 (43.3%) | 185  (56.7% [51; 62]) | 50 | 45  (90%) | 5  (10% [4; 21]) |

^a^ 95% confidence intervals for proportions were calculated using the recommended method by Altman et al. [28]

^b^ Comparison of all groups (live female worms vs. dead female worms): p = 0.4203 (Proc Genmod, SAS®)

^c^ Comparison of all groups (live male worms vs. dead male worms): p = 0.954 (Proc Genmod, SAS®)

*Supplementary table 3a. Embryogenesis (PP)*

| Treatment | No. of patients/  nodules | No. of living female worms | | | | | |
| --- | --- | --- | --- | --- | --- | --- | --- |
|  |  |  | Embryogenesis | | | | |
|  |  | All | Judgement not possible*** | Uterus empty | Oocytes only | Degenerated embryogenesis only | Normal embryogenesis^a, b^ |
| **IVM**  **annual** | 52/152 | 147 | 16 | 32 | 73 | 11  (8.4% [5; 14]) | 15  (11.5% [7; 18]) |
| **IVM semiannual** | 47/142 | 161 | 25 | 28 | 69 | 18  (13.2% [9; 20]) | 21  (15.4% [10; 22]) |
| **IVM + ALB annual** | 55/141 | 160 | 16 | 30 | 63 | 19  (13.2% [9; 20]) | 32  (22.2% [16; 30]) |
| **IVM + ALB semiannual** | 42/128 | 127** | 17 | 26 | 53 | 11  (10% [6; 17]) | 19  (17.3% [11; 25]) |
| All | 196/563* | 595 | 74 | 116 | 375 | 59  (11.3% [9; 14]) | 87  (16.7% [14; 20]) |

*The nodules from 1 patient out of 197 could not be analysed because no oncho nodules or no worm section could be found (IVM + ALB semiannual N = 1), 52 nodules out of 615 nodules analysed were not evaluable (IVM annual N = 8, IVM semiannual N = 9, IVM + ALB annual N = 21, IVM + ALB semiannual N = 14)

** In one live female worm judgement of embryogenesis was possible but could not be assigned to one of the embyrogenic stages as the worm was neoplastic. Therefore the described stages sum up to 126 instead of 127. The worm has been analysed as worm with no normal embryogenesis.

*** In 74 out of the 595 live female worms the judgement of the embryogenesis was not possible for example due to bad quality of the slide, the uterus not being truncated, indefinablecontents of the uterus or folded worm sections.

^a^ 95% confidence intervals for proportions were calculated using the recommended method by Altman et al. [28]

^b^ Comparison of all groups (live female worms with normal embryogenesis vs. all other live female worms with evaluated embryogenesis): p = 0.1722 (Proc Genmod, SAS®)

*Supplementary table 3b. Embryogenesis (ITT, no previous MDA)*

| Treatment | No. of patients/  nodules | No. of living female worms | | | | | |
| --- | --- | --- | --- | --- | --- | --- | --- |
|  |  |  | Embryogenesis | | | | |
|  |  | All | Judgement not possible | Uterus empty | Oocytes only | Degenerated embryogenesis only | Normal embryogenesis^a, b^ |
| **IVM**  **annual** | 14/40 | 47 | 6 | 13 | 18 | 3  (7.3% [3; 19]) | 7  (17.1% [9; 31]) |
| **IVM semiannual** | 17/65 | 52 | 8 | 10 | 26 | 3  (6.8% [2; 18]) | 5  (11.4% [5; 24]) |
| **IVM + ALB annual** | 14/29 | 35 | 3 | 8 | 14 | 3  (9.4% [3; 24]) | 7  (21.9% [11; 39]) |
| **IVM + ALB semiannual** | 10/23 | 24 | 4 | 5 | 10 | 0  (0% [0; 16]) | 5  (25% [11; 47]) |
| All | 55/157 | 158 | 21 | 36 | 68 | 9  (6.6% [3; 12]) | 24  (17.5% [12; 25]) |

^a^ 95% confidence intervals for proportions were calculated using the recommended method by Altman et al. [28]

^b^ Comparison of all groups (live female worms with normal embryogenesis vs. all other live female worms with evaluated embryogenesis): p = 0.6877 (Proc Genmod, SAS®)

*Supplementary table 3c. Embryogenesis (PP, no previous MDA)*

| Treatment | No. of patients/  nodules | No. of living female worms | | | | | |
| --- | --- | --- | --- | --- | --- | --- | --- |
|  |  |  | Embryogenesis | | | | |
|  |  | All | Judgement not possible | Uterus empty | Oocytes only | Degenerated embryogenesis only | Normal embryogenesis |
| **IVM**  **annual** | 13/38 | 44 | 6 | 12 | 16 | 3 (7.9% [3; 21}) | 7 (18.4% [9; 33]) |
| **IVM semiannual** | 15/60 | 49 | 8 | 10 | 24 | 3 (7.3% [3; 19]) | 4 (9.8% [4; 23]) |
| **IVM + ALB annual** | 11/22 | 32 | 2 | 7 | 13 | 3 (10% [3; 26]) | 7 (23.3% [12; 41]) |
| **IVM + ALB semiannual** | 6/18 | 16 | 4 | 4 | 4 | 0 (0% [0; 24]) | 4 (33.3% [14; 61]) |
| All | 45/138 | 141 | 20 | 33 | 90 | 9 (7.4% [4; 14]) | 22 (18.2% [12; 26]) |

^a^ 95% confidence intervals for proportions were calculated using the recommended method by Altman et al. [28]

^b^ Comparison of all groups (live female worms with normal embryogenesis vs. all other live female worms with evaluated embryogenesis): p = 0.4134 (Proc Genmod, SAS®)

*Supplementary table 4a. Free MF in nodules (ITT)*

| Treatment | No. of patients/ nodules | No. of nodules with live female worms or intact MF** | |
| --- | --- | --- | --- |
|  |  | All | With intact MF^a,b^ |
| **IVM annual** | 54/155 | 91 | 7 (7.7% [4; 15]) |
| **IVM semiannual** | 55/165 | 98 | 6 (6.1% [3; 13]) |
| **IVM + ALB annual** | 59/157 | 102 | 14 (13.7% [8; 22]) |
| **IVM + ALB semiannual** | 48/140 | 78 | 9 (11.5% [6; 20]) |
| All | 216/617* | 369 | 36 (9.8% [7; 13]) |

* The nodules from 2 out of 218 patients could not be analysed because no oncho nodules or no worm section could be found (IVM + ALB semiannual N = 2), 60 nodules out of 677 nodules analysed were not evaluable (IVM annual N = 8, IVM semiannual N = 14, IVM + ALB annual N = 22, IVM + ALB semiannual N = 16)

** In the slides of two nodules only dead worms but free intact MF could be detected

^a^ 95% confidence intervals for proportions were calculated using the recommended method by Altman et al. [28]

^b^ Comparison of all groups (Nodules with intact MF vs. all other evaluable nodules): p = 0.3731 (Proc Genmod, SAS®)

*Supplementary table 4b. Free MF in nodules (PP)*

| Treatment | No. of patients/ nodules | No. of nodules with live female worms or intact MF | |
| --- | --- | --- | --- |
|  |  | All | With intact MF^a,b^ |
| **IVM annual** | 52/152 | 88 | 7 (8% [4; 16]) |
| **IVM semiannual** | 47/142 | 84 | 6 (7.1% [3; 15]) |
| **IVM + ALB annual** | 55/141 | 91 | 9 (9.9% [5; 18]) |
| **IVM + ALB semiannual** | 42/128 | 69 | 9 (13% [7; 23]) |
| All | 196/563* | 332 | 31 (9.3% [7; 13]) |

*The nodules from 1 patient out of 197 could not be analysed because no oncho nodules or no worm section could be found (IVM + ALB semiannual N = 1), 52 nodules out of 615 nodules analysed were not evaluable (IVM annual N = 8, IVM semiannual N = 9, IVM + ALB annual N = 21, IVM + ALB semiannual N = 14)

^a^ 95% confidence intervals for proportions were calculated using the recommended method by Altman et al. [28]

^b^ Comparison of all groups (Nodules with intact MF vs. all other evaluable nodules): p = 0.6326 (Proc Genmod, SAS®)

*Supplementary table 5a. Sperms (ITT)*

| Treatment | No. of  patients/  nodules | No. of female worms | | No. of male worms | | |
| --- | --- | --- | --- | --- | --- | --- |
|  |  | Live | With sperms in the uterus^a,b^ | Live | Judgement not possible | With sperms^a,c^ |
| **IVM annual** | 54/155 | 151 | 26 (17.2% [12; 24]) | 50 | 2 | 44 (91.7% [80; 97]) |
| **IVM semiannual** | 55/165 | 182 | 38 (20.9% [16; 27]) | 47 | 2 | 43 (95.6% [85; 99]) |
| **IVM + ALB annual** | 59/157 | 171 | 51 (29.8% [23; 37]) | 50 | 1 | 46 (93.9% [83; 98]) |
| **IVM + ALB semiannual** | 48/140 | 142 | 31 (21.8% [16, 29]) | 41 | 0 | 40 (97.6% [87; 100]) |
| All | 216/617* | 646 | 146 (22.6% [20; 26]) | 188 | 5 | 173 (94.5% [90; 97]) |

* The nodules from 2 out of 218 patients could not be analysed because no oncho nodules or no worm section could be found (IVM + ALB semiannual N = 2), 60 nodules out of 677 nodules analysed were not evaluable (IVM annual N = 8, IVM semiannual N = 14, IVM + ALB annual N = 22, IVM + ALB semiannual N = 16)

^a^ 95% confidence intervals for proportions were calculated using the recommended method by Altman et al. [28]

^b^ Comparison of all groups (live female worms with sperms in the uterus vs. all other live female worms): p = 0.1176 (Proc Genmod, SAS®)

^c^ Comparison of all groups (live male worms with sperms vs. all other live male worms): p = 0.5218 (Proc Genmod, SAS®)

*Supplementary table 5b. Sperms (PP)*

| Treatment | No. of  patients/  nodules | No. of female worms | | No. of male worms | | |
| --- | --- | --- | --- | --- | --- | --- |
|  |  | Live | With sperms in the uterus^a,b^ | Live | Judgement not possible | With sperms^a,c^ |
| **IVM annual** | 52/152 | 147 | 26 (17.7% [12; 25]) | 48 | 2 | 42 (91.3% [80; 97]) |
| **IVM semiannual** | 47/142 | 161 | 36 (22.4% [17; 29]) | 43 | 2 | 39 (95.1% [84; 99]) |
| **IVM + ALB annual** | 55/141 | 160 | 48 (30% [23; 38]) | 46 | 1 | 42 (93.3% [82; 98]) |
| **IVM + ALB semiannual** | 42/128 | 127 | 31 (24.4% [18; 33]) | 40 | 0 | 39 (97.5% [87; 100]) |
| All | 196/563* | 595 | 141 (23.7% [20; 27]) | 177 | 5 | 162 (94.2% [90; 97]) |

*The nodules from 1 patient out of 197 could not be analysed because no oncho nodules or no worm section could be found (IVM + ALB semiannual N = 1), 52 nodules out of 615 nodules analysed were not evaluable (IVM annual N = 8, IVM semiannual N = 9, IVM + ALB annual N = 21, IVM + ALB semiannual N = 14)

^a^ 95% confidence intervals for proportions were calculated using the recommended method by Altman et al. [28]

^b^ Comparison of all groups (live female worms with sperms in the uterus vs. all other live female worms): p = 0.1768 (Proc Genmod, SAS®)

^c^ Comparison of all groups (live male worms with sperms vs. all other live male worms): p = 0.5062 (Proc Genmod, SAS®)

*Supplementary table 6a. Microfiladermia (PP)*

|  |  | **IVM**  **annual** | **IVM semiannual** | **IVM + ALB annual** | **IVM + ALB semiannual** | ***p-value*** |
| --- | --- | --- | --- | --- | --- | --- |
| **Baseline** | N | 54 | 50 | 60 | 45 |  |
| MF positive | N (%) | 54 (100%) | 50 (100%) | 60 (100%) | 45 (100%) |  |
| MF/mg skin | Median | 4.5 | 2.8 | 4.5 | 3.9 |  |
|  | 95% CI of the median^b^ | [2.2; 9.4] | [1; 5.3] | [2; 7.7] | [1.1; 6.9] |  |
|  | Geometric mean^c^ | 5.7 | 3.5 | 5.3 | 4.6 |  |
|  | Min - Max | 0.2 - 69.5 | 0.1 - 113 | 0.1 - 367 | 0.2 - 159 |  |
|  | 25^th^; 75^th^ percentiles | 1.4; 15.9 | 0.5; 10.1 | 0.8; 12.8 | 0.7; 10.8 | p = 0.332^e^ |
| **6 months** | N | 52 | 50 | 58 | 45 |  |
| MF positive | N (%) | 22 (42.3%) | 18 (36%) | 23 (39.7%) | 13 (28.9%) | p = 0.561^d^ |
|  | [95%CI]^a^ | [29.9; 55.8] | [24.1; 49.9] | [28.1; 52.5] | [17.7; 43.4] |  |
| MF/mg skin | Median | 0 | 0 | 0 | 0 |  |
|  | 95% CI of the median^b^ | [0; 0.25] | [0; 0.09] | [0; 0.17] | [0; 0] |  |
|  | Geometric mean^c^ | 0.27 | 0.26 | 0.33 | 0.2 |  |
|  | Min - Max | 0 - 4.2 | 0 - 4.6 | 0 - 13.9 | 0 - 3.3 |  |
|  | 25^th^; 75^th^ percentiles | 0; 0.3 | 0; 0.4 | 0; 0.5 | 0; 0.1 | p = 0.577^e^ |
| *p-value (comparison to baseline)^f^* | | p < 0.001 | p < 0.001 | p < 0.001 | p < 0.001 |  |
| **18 months** | N | 52 | 50 | 57 | 45 |  |
| MF positive | N (%) | 12 (23.1%) | 3 (6%) | 11 (19.3%) | 7 (15.6%) | p = 0.087^d^ |
|  | [95%CI]^a^ | [13.7; 36.1] | [2.1; 16.2] | [11.1; 31.3] | [7.7; 28.8] |  |
| MF/mg skin | Median | 0 | 0 | 0 | 0 |  |
|  | 95% CI of the median^b^ | [0; 0] | [0; 0] | [0; 0] | [0; 0] |  |
|  | Geometric mean^c^ | 0.09 | 0.02 | 0.12 | 0.07 |  |
|  | Min - Max | 0 - 3.7 | 0 - 0.7 | 0 - 4.2 | 0 - 1.6 |  |
|  | 25^th^; 75^th^ percentiles | 0; 0 | 0; 0 | 0; 0 | 0; 0 | p = 0.121^e^ |
| *p-value (comparison to baseline)^f^* | | p < 0.001 | p < 0.001 | p < 0.001 | p < 0.001 |  |
| **36 months** | N | 54 | 50 | 60 | 45 |  |
| MF positive | N (%) | 20 (37%) | 14 (28%) | 22 (36.7%) | 9 (20%) | p = 0.201^d^ |
|  | [95%CI]^a^ | [25.4; 50.4] | [17.5; 41.7] | [25.6; 49.3] | [10.9; 33.8] |  |
| MF/mg skin | Median | 0 | 0 | 0 | 0 |  |
|  | 95% CI of the median^b^ | [0; 0.05] | [0; 0] | [0; 0] | [0; 0] |  |
|  | Geometric mean^c^ | 0.2 | 0.13 | 0.22 | 0.16 |  |
|  | Min - Max | 0 - 6.3 | 0 - 1.8 | 0 - 11.1 | 0 - 6.1 |  |
|  | 25^th^; 75^th^ percentiles | 0; 0.3 | 0; 0.1 | 0; 0.1 | 0; 0 | p = 0.289^e^ |
| *p-value (comparison to baseline)^f^* | | p < 0.001 | p < 0.001 | p < 0.001 | p < 0.001 |  |

^a^ Confidence intervals for proportions were calculated using the recommended method by Altman et al. [28]

^b^ Confidence intervals for the median were calculated using bootstrapping

^c^ the geometric mean was calculated by adding 1 to the original MF-values and subtracting 1 from the final result

^d^ Fisher’s exact test

^e^ Kruskal-Wallis-test

^f^ Wilcoxon signed rank test

*Supplementary table 6b. Microfiladermia (ITT, no previous MDA)*

|  |  | **IVM**  **annual** | **IVM semiannual** | **IVM + ALB annual** | **IVM + ALB semiannual** | ***p-value*** |
| --- | --- | --- | --- | --- | --- | --- |
| **Baseline** | N | 19 | 22 | 18 | 17 |  |
| MF positive | N (%) | 19 (100%) | 22 (100%) | 18 (100%) | 17 (100%) |  |
| MF/mg skin | Median | 10.5 | 6 | 9.1 | 3.4 |  |
|  | 95% CI of the median^b^ | [6.2; 25.4] | [3; 26.5] | [4.6; 24.5] | [1.2; 9.9] |  |
|  | Geometric mean^c^ | 11.2 | 8.8 | 11.8 | 6.0 |  |
|  | Min - Max | 0.3 - 69.5 | 0.1 - 113 | 0.2 - 152 | 0.6 - 77.8 |  |
|  | 25^th^; 75^th^ percentiles | 4.9; 31 | 3.1; 28.5 | 4.6; 33.5 | 1.2; 9.9 | p = 0.205^e^ |
| **6 months** | N | 16 | 22 | 17 | 15 |  |
| MF positive | N (%) | 9 (56.3%) | 8 (36.4%) | 5 (29.4%) | 2 (13.3%) | p = 0.088^d^ |
|  | [95%CI]^a^ | [33.2; 76.9] | [19.7; 57] | [13.3; 53.1] | [3.7; 37.9] |  |
| MF/mg skin | Median | 0.2 | 0 | 0 | 0 |  |
|  | 95% CI of the median^b^ | [0; 0.6] | [0; 0.13] | [0; 0.3] | [0; 0] |  |
|  | Geometric mean^c^ | 0.4 | 0.19 | 0.12 | 0.12 |  |
|  | Min - Max | 0 - 4.2 | 0 - 4.6 | 0 - 0.8 | 0 - 2.4 |  |
|  | 25^th^; 75^th^ percentiles | 0; 0.6 | 0; 0.2 | 0; 0.3 | 0; 0 | p = 0.11^e^ |
| *p-value (comparison to baseline)^f^* | | p = 0.001 | p < 0.001 | p < 0.001 | p = 0.001 |  |
| **18 months** | N | 15 | 22 | 15 | 14 |  |
| MF positive | N (%) | 5 (33.3%) | 1 (4.5%) | 3 (20.0%) | 1 (7.1%) | p = 0.085^d^ |
|  | [95%CI]^a^ | [15.2; 58.3] | [0.8; 21.8] | [7; 45.2] | [1.3; 31.5] |  |
| MF/mg skin | Median | 0 | 0 | 0 | 0 |  |
|  | 95% CI of the median^b^ | [0; 0.1] | [0; 0] | [0; 0] | [0; 0] |  |
|  | Geometric mean^c^ | 0.17 | 0.02 | 0.06 | 0.01 |  |
|  | Min - Max | 0 - 3.7 | 0 - 0.5 | 0 - 0.5 | 0 - 0.2 |  |
|  | 25^th^; 75^th^ percentiles | 0; 0.1 | 0; 0 | 0; 0 | 0; 0 | p = 0.098^e^ |
| *p-value (comparison to baseline)^f^* | | p = 0.001 | p < 0.001 | p = 0.001 | p = 0.001 |  |
| **36 months** | N | 15 | 19 | 17 | 12 |  |
| MF positive | N (%) | 8 (53.3%) | 7 (36.8%) | 8 (47.1%) | 1 (8.3%) | p = 0.075^d^ |
|  | [95%CI]^a^ | [30.1; 75.2] | [19.1; 59] | [26.2; 69] | [1.5; 35.4] |  |
| MF/mg skin | Median | 0.1 | 0 | 0 | 0 |  |
|  | 95% CI of the median^b^ | [0; 0.4] | [0; 0.06] | [0; 0.3] | [0; 0] |  |
|  | Geometric mean^c^ | 0.21 | 0.13 | 0.32 | 0.01 |  |
|  | Min - Max | 0 - 1.0 | 0 - 1.8 | 0 - 7.5 | 0 - 0.1 |  |
|  | 25^th^; 75^th^ percentiles | 0; 0.4 | 0; 0.2 | 0; 0.3 | 0; 0 | p = 0.056^e^ |
| *p-value (comparison to baselinef^e^* | | p = 0.001 | p < 0.001 | p < 0.001 | p = 0.002 |  |

^a^ Confidence intervals for proportions were calculated using the recommended method by Altman et al. [28]

^b^ Confidence intervals for the median were calculated using bootstrapping

^c^ the geometric mean was calculated by adding 1 to the original MF-values and subtracting 1 from the final result

^d^ Fisher’s exact test

^e^ Kruskal-Wallis-test

^f^ Wilcoxon signed rank test

*Supplementary table 6c. Microfiladermia (PP, no previous MDA)*

|  |  | **IVM**  **annual** | **IVM semiannual** | **IVM + ALB annual** | **IVM + ALB semiannual** | ***p-value*** |
| --- | --- | --- | --- | --- | --- | --- |
| **Baseline** | N | 14 | 16 | 14 | 8 |  |
| MF positive | N (%) | 14 (100%) | 16 (100%) | 14 (100%) | 8 (100%) |  |
| MF/mg skin | Median | 17.8 | 6 | 8.3 | 4.1 |  |
|  | 95% CI of the median^b^ | [9.3; 36.8] | [2.7; 21.9] | [3.8; 17.8] | [0.9; 22.3] |  |
|  | Geometric mean^c^ | 17.3 | 7.7 | 9.8 | 5.6 |  |
|  | Min - Max | 2.6 - 69.5 | 0.1 - 113 | 0.2 - 152 | 0.6 - 77.8 |  |
|  | 25^th^; 75^th^ percentiles | 9.3; 35.4 | 2.7; 24.5 | 3.8; 17.8 | 1.1; 16.1 | p = 0.163^e^ |
| **6 months** | N | 12 | 16 | 13 | 8 |  |
| MF positive | N (%) | 8 (66.7%) | 6 (37.5%) | 2 (15.4%) | 1 (12.5%) | p = 0.03^d^ |
|  | [95%CI]^a^ | [39.1; 86.2] | [18.5; 61.4] | [4.3; 42.2] | [2.2; 47.1] |  |
| MF/mg skin | Median | 0.3 | 0 | 0 | 0 |  |
|  | 95% CI of the median^b^ | [0; 1.4] | [0; 0.3] | [0; 0] | [0; 1.2] |  |
|  | Geometric mean^c^ | 0.48 | 0.25 | 0.07 | 0.17 |  |
|  | Min - Max | 0 - 4.2 | 0 - 4.6 | 0 - 0.8 | 0 - 2.4 |  |
|  | 25^th^; 75^th^ percentiles | 0; 0.9 | 0; 0.3 | 0; 0 | 0; 0 | p = 0.05^e^ |
| *p-value (comparison to baseline)^f^* | | p = 0.002 | p < 0.001 | p = 0.001 | p = 0.012 |  |
| **18 months** | N | 12 | 16 | 13 | 8 |  |
| MF positive | N (%) | 4 (33.3%) | 1 (6.3%) | 3 (23.1%) | 1 (12.5%) | p = 0.292^d^ |
|  | [95%CI]^a^ | [13.8; 60.9] | [1.1; 28.3] | [8.2; 50.3] | [2.2; 47.1] |  |
| MF/mg skin | Median | 0 | 0 | 0 | 0 |  |
|  | 95% CI of the median^b^ | [0; 0.3] | [0; 0] | [0; 0.1] | [0; 0] |  |
|  | Geometric mean^c^ | 0.21 | 0.03 | 0.07 | 0.02 |  |
|  | Min - Max | 0 - 3.7 | 0 - 0.5 | 0 - 0.5 | 0 - 0.2 |  |
|  | 25^th^; 75^th^ percentiles | 0; 0.2 | 0; 0 | 0; 0 | 0; 0 | p = 0.325^e^ |
| *p-value (comparison to baseline)^f^* | | p = 0.002 | p < 0.001 | p = 0.001 | p = 0.012 |  |
| **36 months** | N | 14 | 16 | 14 | 8 |  |
| MF positive | N (%) | 7 (50.0%) | 7 (43.8%) | 6 (42.9%) | 1 (12.5%) | p = 0.375^d^ |
|  | [95%CI]^a^ | [26.8; 73.2] | [23.1; 66.8] | [21.4; 67.4] | [2.2; 47.1] |  |
| MF/mg skin | Median | 0 | 0 | 0 | 0 |  |
|  | 95% CI of the median^b^ | [0; 0.4] | [0; 0.2] | [0; 0.2] | [0; 0.04] |  |
|  | Geometric mean^c^ | 0.2 | 0.16 | 0.28 | 0.01 |  |
|  | Min - Max | 0 - 1 | 0 - 1.8 | 0 - 7.5 | 0 - 0.1 |  |
|  | 25^th^; 75^th^ percentiles | 0; 0.4 | 0; 0.2 | 0; 0.2 | 0; 0 | p = 0.265^e^ |
| *p-value (comparison to baseline)^f^* | | p = 0.001 | p = 0.001 | p = 0.001 | p = 0.012 |  |

^a^ Confidence intervals for proportions were calculated using the recommended method by Altman et al. [28]

^b^ Confidence intervals for the median were calculated using bootstrapping

^c^ the geometric mean was calculated by adding 1 to the original MF-values and subtracting 1 from the final result

^d^ Fisher’s exact test

^e^ Kruskal-Wallis-test

^f^ Wilcoxon signed rank test

*Supplementary table 6d. Microfiladermia (PP, annual vs. semiannual treatment)*

|  |  | **Annual**  **treatment** | **Semiannual treatment** | ***p-value*** |
| --- | --- | --- | --- | --- |
| **Baseline** | N | 114 | 95 |  |
| MF positive | N (%) | 114 (100%) | 95 (100%) |  |
| MF/mg skin | Median | 4.5 | 3.3 |  |
|  | 95% CI of the median^b^ | [2.6; 7.4] | [1.4; 5.1] |  |
|  | Geometric mean^c^ | 5.5 | 4.0 |  |
|  | Min - Max | 0.1 - 367 | 0.1 - 159 |  |
|  | 25^th^; 75^th^ percentiles | 1.1; 14.8 | 0.5; 10.4 | p = 0.119^e^ |
| **6 months** | N | 110 | 95 |  |
| MF positive | N (%) | 45 (40.9%) | 31 (32.6%) | p = 0.248^d^ |
|  | [95%CI]^a^ | [32.2; 50.3] | [24; 42.6] |  |
| MF/mg skin | Median | 0 | 0 |  |
|  | 95% CI of the median^b^ | [0; 0.1] | [0; 0] |  |
|  | Geometric mean^c^ | 0.3 | 0.23 |  |
|  | Min - Max | 0 - 13.9 | 0 - 4.6 |  |
|  | 25^th^; 75^th^ percentiles | 0; 0.4 | 0; 0.3 | p = 0.233^e^ |
|  | *p-value (comparison to baseline)^f^* | p < 0.001 | p < 0.001 |  |
| **18 months** | N | 109 | 95 |  |
| MF positive | N (%) | 23 (21.1%) | 10 (10.5%) | p = 0.056^d^ |
|  | [95%CI]^a^ | [14.5; 29.7] | [5.8; 18.3] |  |
| MF/mg skin | Median | 0 | 0 |  |
|  | 95% CI of the median^b^ | [0; 0] | [0; 0] |  |
|  | Geometric mean^c^ | 0.1 | 0.05 |  |
|  | Min - Max | 0 - 4.2 | 0 - 1.6 |  |
|  | 25^th^; 75^th^ percentiles | 0; 0 | 0; 0 | **p = 0.04^e^** |
|  | *p-value (comparison to baseline)^f^* | p < 0.001 | p < 0.001 |  |
| **36 months** | N | 114 | 95 |  |
| MF positive | N (%) | 42 (36.8%) | 23 (24.2%) | p = 0.053^d^ |
|  | [95%CI]^a^ | [28.6; 46] | [16.7; 33.7] |  |
| MF/mg skin | Median | 0 | 0 |  |
|  | 95% CI of the median^b^ | [0; 0] | [0; 0] |  |
|  | Geometric mean^c^ | 0.21 | 0.14 |  |
|  | Min - Max | 0 - 11.1 | 0 - 6.1 |  |
|  | 25^th^; 75^th^ percentiles | 0; 0.2 | 0; 0 | p = 0.069^e^ |
|  | *p-value (comparison to baseline)^f^* | p < 0.001 | p < 0.001 |  |

^a^ Confidence intervals for proportions were calculated using the recommended method by Altman et al. [28]

^b^ Confidence intervals for the median were calculated using bootstrapping

^c^ the geometric mean was calculated by adding 1 to the original MF-values and subtracting 1 from the final result

^d^ Fisher’s exact test

^e^ Kruskal-Wallis-test

^f^ Wilcoxon signed rank test

*Supplementary table 6e. Microfiladermia (ITT, annual vs. semiannual treatment, no previous MDA)*

|  |  | **Annual**  **treatment** | **Semiannual treatment** | ***p-value*** |
| --- | --- | --- | --- | --- |
| **Baseline** | N | 37 | 39 |  |
| MF positive | N (%) | 37 (100%) | 39 (100%) |  |
| MF/mg skin | Median | 9.9 | 5.4 |  |
|  | 95% CI of the median^b^ | [6.3; 17.1] | [3.1; 10.1] |  |
|  | Geometric mean^c^ | 11.5 | 6.9 |  |
|  | Min - Max | 0.2 - 152 | 0.1 - 113 |  |
|  | 25^th^; 75^th^ percentiles | 4.9; 31.0 | 1.7; 22.3 | p = 0.081^e^ |
| **6 months** | N | 33 | 37 |  |
| MF positive | N (%) | 14 (42.4%) | 10 (27%) | p = 0.212^d^ |
|  | [95%CI]^a^ | [27.2; 59.2] | [15.4; 43] |  |
| MF/mg skin | Median | 0 | 0 |  |
|  | 95% CI of the median^b^ | [0; 0.3] | [0; 0] |  |
|  | Geometric mean^c^ | 0.24 | 0.16 |  |
|  | Min - Max | 0 - 4.2 | 0 - 4.6 |  |
|  | 25^th^; 75^th^ percentiles | 0; 0.3 | 0; 0.1 | p = 0.158^e^ |
|  | *p-value (comparison to baseline)^e^* | p < 0.001 | p < 0.001 |  |
| **18 months** | N | 30 | 36 |  |
| MF positive | N (%) | 8 (26.7%) | 2 (5.6%) | **p = 0.035^d^** |
|  | [95%CI]^a^ | [14.2; 44.4] | [1.5; 18.1] |  |
| MF/mg skin | Median | 0 | 0 |  |
|  | 95% CI of the median^b^ | [0; 0] | [0; 0] |  |
|  | Geometric mean^c^ | 0.11 | 0.02 |  |
|  | Min - Max | 0 - 3.7 | 0 - 0.5 |  |
|  | 25^th^; 75^th^ percentiles | 0; 0.1 | 0; 0 | **p = 0.021^e^** |
|  | *p-value (comparison to baseline)^e^* | p < 0.001 | p < 0.001 |  |
| **36 months** | N | 32 | 31 |  |
| MF positive | N (%) | 16 (50%) | 8 (25.8%) | p = 0.07^d^ |
|  | [95%CI]^a^ | [33.6; 66.4] | [13.7; 43.2] |  |
| MF/mg skin | Median | 0 | 0 |  |
|  | 95% CI of the median^b^ | [0; 0.2] | [0; 0] |  |
|  | Geometric mean^c^ | 0.26 | 0.0 |  |
|  | Min - Max | 0 - 7.5 | 0 - 1.8 |  |
|  | 25^th^; 75^th^ percentiles | 0; 0.4 | 0; 0.1 | **p = 0.023^e^** |
|  | *p-value (comparison to baseline)^e^* | p < 0.001 | p < 0.001 |  |

^a^ Confidence intervals for proportions were calculated using the recommended method by Altman et al. [28]

^b^ Confidence intervals for the median were calculated using bootstrapping

^c^ the geometric mean was calculated by adding 1 to the original MF-values and subtracting 1 from the final result

^d^ Fisher’s exact test

^e^ Kruskal-Wallis-test

^f^ Wilcoxon signed rank test

*Supplementary table 6f. Microfiladermia (PP, annual vs. semiannual treatment, no previous MDA)*

|  |  | **Annual**  **treatment** | **Semiannual treatment** | ***p-value*** |
| --- | --- | --- | --- | --- |
| **Baseline** | N | 28 | 24 |  |
| MF positive | N (%) | 28 (100%) | 24 (100%) |  |
| MF/mg skin | Median | 11.1 | 5.5 |  |
|  | 95% CI of the median^b^ | [7.7; 25.0] | [2.9; 13.7] |  |
|  | Geometric mean^c^ | 13 | 6.9 |  |
|  | Min - Max | 0.2 - 152 | 0.1 - 113 |  |
|  | 25^th^; 75^th^ percentiles | 5.5; 33.2 | 1.9; 22.1 | p = 0.081^e^ |
| **6 months** | N | 25 | 24 |  |
| MF positive | N (%) | 10 (40.0%) | 7 (29.2%) | p = 0.551^d^ |
|  | [95%CI]^a^ | [23.4; 59.3] | [14.9; 49.2] |  |
| MF/mg skin | Median | 0 | 0 |  |
|  | 95% CI of the median^b^ | [0; 0.3] | [0; 0.1] |  |
|  | Geometric mean^c^ | 0.25 | 0.22 |  |
|  | Min - Max | 0 - 4.2 | 0 - 4.6 |  |
|  | 25^th^; 75^th^ percentiles | 0; 0.3 | 0; 0.2 | p = 0.495^e^ |
|  | *p-value (comparison to baseline)^f^* | p < 0.001 | p < 0.001 |  |
| **18 months** | N | 25 | 24 |  |
| MF positive | N (%) | 7 (28.0%) | 2 (8.3%) | p = 0.138^d^ |
|  | [95%CI]^a^ | [14.3; 47.6] | [2.3; 25.8] |  |
| MF/mg skin | Median | 0 | 0 |  |
|  | 95% CI of the median^b^ | [0; 0] | [0; 0] |  |
|  | Geometric mean^c^ | 0.13 | 0.02 |  |
|  | Min - Max | 0 - 3.7 | 0 - 0.5 |  |
|  | 25^th^; 75^th^ percentiles | 0; 0.1 | 0; 0 | p = 0.086^e^ |
|  | *p-value (comparison to baseline)^f^* | p < 0.001 | p < 0.001 |  |
| **36 months** | N | 28 | 24 |  |
| MF positive | N (%) | 13 (46.4%) | 8 (33.3%) | p = 0.403^d^ |
|  | [95%CI]^a^ | [29.5; 64.2] | [18.0; 53.3] |  |
| MF/mg skin | Median | 0 | 0 |  |
|  | 95% CI of the median^b^ | [0; 0.2] | [0; 0.1] |  |
|  | Geometric mean^c^ | 0.24 | 0.11 |  |
|  | Min - Max | 0 - 7.5 | 0 - 1.8 |  |
|  | 25^th^; 75^th^ percentiles | 0; 0.3 | 0; 0.1 | p = 0.207^e^ |
|  | *p-value (comparison to baseline)^f^* | p < 0.001 | p < 0.001 |  |

^a^ Confidence intervals for proportions were calculated using the recommended method by Altman et al. [28]

^b^ Confidence intervals for the median were calculated using bootstrapping

^c^ the geometric mean was calculated by adding 1 to the original MF-values and subtracting 1 from the final result

^d^ Fisher’s exact test

^e^ Kruskal-Wallis-test

^f^ Wilcoxon signed rank test

*Supplementary table 7. Adverse events*

|  |  | **IVM**  **annual** | **IVM semiannual** | **IVM + ALB annual** | **IVM + ALB semiannual** |
| --- | --- | --- | --- | --- | --- |
| **0 months** |  |  |  |  |  |
| No. of patients with AEs/ total number of patients |  | 43/68 (63.2%) | 48/67 (71.6%) | 50/70 (71.4%) | 45/66 (68.2%) |
| No. of AEs per patient | N | 68 | 67 | 70 | 66 |
|  | Mean ± SD | 1.5 ± 1.6 | 1.4 ± 1.4 | 1.6 ± 1.6 | 1.6 ± 1.6 |
|  | Min - Max | 0 - 6 | 0 - 6 | 0 - 7 | 0 - 7 |
|  | Median | 1 | 1 | 1 | 1 |
|  | 25^th^; 75^th^ percentiles | 0; 3 | 0; 2 | 0; 2 | 0; 3 |
| No. of AEs in total | N | 105 | 97 | 110 | 103 |
|  | Grade 1 | 91 (86.7%) | 84 (86.6%) | 104 (94.5%) | 94 (91.3%) |
|  | Grade 2 | 13 (12.4%) | 11 (11.3%) | 6 (5.5%) | 8 (7.8%) |
|  | Grade 3 | 1 (1%) | 2 (2.1%) | 0 (0%) | 1 (1%) |
| AE relation to treatment | N | 97 | 96 | 107 | 101 |
|  | definite | 0 (0%) | 2 (2.1%) | 0 (0%) | 0 (0%) |
|  | probable | 47 (48.5%) | 45 (46.9%) | 59 (55.1%) | 49 (48.5%) |
|  | possible | 33 (34%) | 28 (29.2%) | 30 (28%) | 34 (33.7%) |
|  | remote | 10 (10.3%) | 9 (9.4%) | 12 (11.2%) | 9 (8.9%) |
|  | not related | 7 (7.2%) | 12 (12.5%) | 6 (5.6%) | 9 (8.9%) |
| Duration of AEs (days) | N | 43 | 47 | 50 | 45 |
|  | Mean ± SD | 2.23 ± 1.36 | 1.64 ± 0.94 | 1.9 ± 0.93 | 2.09 ± 1.2 |
|  | Min - Max | 1 - 6 | 1 - 5 | 1 - 4 | 1 - 6 |
|  | Median | 2 | 1 | 2 | 2 |
|  | 25^th^; 75^th^ percentiles | 1; 3 | 1; 2 | 1; 2 | 1; 3 |

|  |  | **IVM**  **annual** | **IVM semiannual** | **IVM + ALB annual** | **IVM + ALB semiannual** |
| --- | --- | --- | --- | --- | --- |
| **6 months** |  |  |  |  |  |
| No. of patients with AEs/ total number of patients |  | 11/62 (17.7%) | 16/66 (24.2%) | 5/66  (7.6%) | 16/63  (25.4%) |
| No. of AEs per patient | N | 62 | 66 | 66 | 63 |
|  | Mean ± SD | 0.2 ± 0.6 | 0.4 ± 0.9 | 0.1 ± 0.3 | 0.3 ± 0.7 |
|  | Min - Max | 0 - 4 | 0 - 4 | 0 - 2 | 0 - 2 |
|  | Median | 0 | 0 | 0 | 0 |
|  | 25th; 75th percentiles | 0; 0 | 0; 0 | 0; 0 | 0; 1 |
| No. of AEs in total | N | 15 | 26 | 6 | 22 |
|  | Grade 1 | 13 (86.7%) | 23 (88.5%) | 6 (100%) | 18 (81.8%) |
|  | Grade 2 | 2 (15.4%) | 3 (11.5%) | 0 (0%) | 4 (18.2%) |
| AE relation to treatment | N | 15 | 24 | 6 | 21 |
|  | definite | 0 (0%) | 1 (4.2%) | 0 (0%) | 0 (0%) |
|  | probable | 0 (0%) | 4 (16.7%) | 0 (0%) | 2 (9.5%) |
|  | possible | 0 (0%) | 11 (45.8%) | 0 (0%) | 18 (85.7%) |
|  | remote | 0 (0%) | 2 (8.3%) | 0 (0%) | 1 (4.8%) |
|  | not related | 15 (100%) | 6 (25%) | 6 (100%) | 0 (0%) |
| Duration of AEs (days) | N | 11 | 16 | 5 | 16 |
|  | Mean ± SD | 1.27 ± 0.47 | 1.69 ± 0.87 | 1.6 ± 0.89 | 1.25 ± 0.45 |
|  | Min - Max | 1 - 2 | 1 - 4 | 1 - 3 | 1 - 2 |
|  | Median | 1 | 1.5 | 1 | 1 |
|  | 25th; 75th percentiles | 1; 2 | 1; 2 | 1; 2 | 1; 1.5 |

|  |  | **IVM**  **annual** | **IVM semiannual** | **IVM + ALB annual** | **IVM + ALB semiannual** |
| --- | --- | --- | --- | --- | --- |
| **12 months** |  |  |  |  |  |
| No. of patients with AEs/ total number of patients |  | 9/66 (13.6%) | 6/62  (9.7%) | 13/67 (19.4%) | 11/62 (17.7%) |
| No. of AEs per patient | N | 66 | 62 | 67 | 62 |
|  | Mean ± SD | 0.2 ± 0.5 | 0.2 ± 0.5 | 0.2 ± 0.5 | 0.2 ± 0.5 |
|  | Min - Max | 0 - 3 | 0 - 3 | 0 - 2 | 0 - 2 |
|  | Median | 0 | 0 | 0 | 0 |
|  | 25^th^; 75^th^ percentiles | 0; 0 | 0; 0 | 0; 0 | 0; 0 |
| No. of AEs in total | N | 12 | 10 | 16 | 14 |
|  | Grade 1 | 11 (91.7%) | 9 (90%) | 16 (100%) | 14 (100%) |
|  | Grade 2 | 1 (8.3%) | 1 (10%) | 0 (0%) | 0 (0%) |
| AE relation to treatment | N | 12 | 10 | 16 | 14 |
|  | probable | 2 (16.7%) | 1 (10%) | 0 (0%) | 0 (0%) |
|  | possible | 8 (66.7%) | 1 (10%) | 12 (75%) | 9 (64.3%) |
|  | remote | 0 (0%) | 4 (40%) | 2 (12.5%) | 4 (28.6%) |
|  | not related | 2 (16.7%) | 4 (40%) | 2 (12.5%) | 1 (7.1%) |
| Duration of AEs (days) | N | 9 | 6 | 13 | 11 |
|  | Mean ± SD | 1.33 ± 0.71 | 1.50 ± 1.22 | 1.08 ± 0.28 | 1.64 ± 0.67 |
|  | Min - Max | 1 - 3 | 1 - 4 | 1 - 2 | 1 - 3 |
|  | Median | 1 | 1 | 1 | 2 |
|  | 25^th^; 75^th^ percentiles | 1; 1 | 1; 1 | 1; 1 | 1; 2 |

|  |  | **IVM**  **annual** | **IVM semiannual** | **IVM + ALB annual** | **IVM + ALB semiannual** |
| --- | --- | --- | --- | --- | --- |
| **18 months** |  |  |  |  |  |
| No. of patients with AEs/ total number of patients |  | 6/62  (9.7%) | 13/64 (20.3%) | 4/64  (6.3%) | 7/60  (11.7%) |
| No. of AEs per patient | N | 62 | 64 | 64 | 60 |
|  | Mean ± SD | 0.1 ± 0.4 | 0.3 ± 0.7 | 0.1 ± 0.2 | 0.2 ± 0.5 |
|  | Min - Max | 0 - 2 | 0 - 4 | 0 - 1 | 0 - 2 |
|  | Median | 0 | 0 | 0 | 0 |
|  | 25^th^; 75^th^ percentiles | 0; 0 | 0; 0 | 0; 0 | 0; 0 |
| No. of AEs in total | N | 8 | 20 | 4 | 11 |
|  | Grade 1 | 8 (100%) | 19 (95%) | 4 (100%) | 8 (72.7%) |
|  | Grade 2 | 0 (0%) | 1 (5%) | 0 (0%) | 3 (27.3%) |
| AE relation to treatment | N | 8 | 20 | 3 | 11 |
|  | definite | 0 (0%) | 3 (15%) | 0 (0%) | 2 (18.2%) |
|  | probable | 0 (0%) | 9 (45%) | 0 (0%) | 6 (54.5%) |
|  | possible | 1 (12.5%) | 6 (30%) | 0 (0%) | 1 (9.1%) |
|  | remote | 1 (12.5%) | 1 (5%) | 1 (33.3%) | 0 (0%) |
|  | not related | 6 (75%) | 1 (5%) | 2 (66.7%) | 2 (18.2%) |
| Duration of AEs (days) | N | 6 | 13 | 4 | 7 |
|  | Mean ± SD | 1 ± 0 | 1.62 ± 0.87 | 1 ± 0 | 1.43 ± 0.53 |
|  | Min - Max | 1 - 1 | 1 - 3 | 1 - 1 | 1 - 2 |
|  | Median | 1 | 1 | 1 | 1 |
|  | 25^th^; 75^th^ percentiles | 1; 1 | 1; 2 | 1; 1 | 1; 2 |

|  |  | **IVM**  **annual** | **IVM semiannual** | **IVM + ALB annual** | **IVM + ALB semiannual** |
| --- | --- | --- | --- | --- | --- |
| **24 months** |  |  |  |  |  |
| No. of patients with AEs/ total number of patients |  | 8/63 (12.7%) | 6/65  (9.2%) | 12/66 (18.2%) | 7/60  (11.7%) |
| No. of AEs per patient | N | 63 | 65 | 66 | 60 |
|  | Mean ± SD | 0.2 ± 0.4 | 0.1 ± 0.4 | 0.2 ± 0.4 | 0.1 ± 0.3 |
|  | Min - Max | 0 - 2 | 0 - 2 | 0 - 2 | 0 - 1 |
|  | Median | 0 | 0 | 0 | 0 |
|  | 25^th^; 75^th^ percentiles | 0; 0 | 0; 0 | 0; 0 | 0; 0 |
| No. of AEs in total | N | 10 | 8 | 13 | 7 |
|  | Grade 1 | 7 (70%) | 8 (100%) | 13 (100%) | 6 (85.7%) |
|  | Grade 2 | 3 (30%) | 0 (0%) | 0 (0%) | 1 (14.3%) |
| AE relation to treatment | N | 10 | 8 | 13 | 7 |
|  | definite | 2 (20%) | 1 (12.5%) | 2 (15.4%) | 1 (14.3%) |
|  | probable | 2 (20%) | 3 (37.5%) | 5 (38.5%) | 4 (57.1%) |
|  | possible | 2 (20%) | 4 (50%) | 4 (30.8%) | 0 (0%) |
|  | remote | 4 (40%) | 0 (0%) | 0 (0%) | 0 (0%) |
|  | not related | 0 (0%) | 0 (0%) | 2 (15.4%) | 2 (28.6%) |
| Duration of AEs (days) | N | 8 | 6 | 12 | 7 |
|  | Mean ± SD | 1.13 ± 0.35 | 1.33 ± 0.52 | 1 ± 0 | 1 ± 0 |
|  | Min - Max | 1 - 2 | 1 - 2 | 1 - 1 | 1 - 1 |
|  | Median | 1 | 1 | 1 | 1 |
|  | 25^th^; 75^th^ percentiles | 1; 1 | 1; 2 | 1; 1 | 1; 1 |
